# Reversing MET‐Mediated Resistance in Oncogene‐Driven NSCLC by MET‐Activated Wnt Condensative Prodrug

**DOI:** 10.1002/advs.202400603

**Published:** 2024-06-13

**Authors:** Na Liu, Xiaoqiang Zheng, Jin Yan, Aimin Jiang, Yu Yao, Wangxiao He

**Affiliations:** ^1^ Department of Medical Oncology The First Affiliated Hospital of Xi'an Jiaotong University Xi'an 710061 P. R. China; ^2^ Institute for Stem Cell & Regenerative Medicine The Second Affiliated Hospital of Xi'an Jiaotong University Xi'an 710004 P. R. China; ^3^ Department of Infectious Diseases The Second Affiliated Hospital of Xi'an Jiaotong University Xi'an 710004 P. R. China; ^4^ Department of Tumor and Immunology in Precision Medical Institute Western China Science and Technology Innovation Port The Second Affiliated Hospital of Xi'an Jiaotong University Xi'an 710004 P. R. China; ^5^ Department of Talent Highland The First Affiliated Hospital of Xi'an Jiao Tong University Xi'an 710061 P. R. China

**Keywords:** drug development, MET amplification, NSCLC, peptide, Wnt inhibitor

## Abstract

The amplification of MET is a major cause of acquired resistance to targeted therapy in EGFR‐mutant non‐small‐cell lung cancer (NSCLC), only to be temporarily restrained by the partial efficacy of MET inhibitors. This study reveals that the MET inhibitor has unexpectedly limited efficacy due to amplified MET triggering a strong positive feedback loop in the Wnt/β‐catenin signaling pathway, allowing optimal functionality even when the MET pathway is suppressed again. To test this conjecture and specifically target the Wnt/β‐catenin pathway, a cleverly designed Wnt condensative pro drug called WntSI is developed using reversible supramolecular self‐assembly driven by liquidliquid phase separation (LLPS). This process involves a MET/pH‐responsive peptide (Tyr‐Pep) and a potent Wnt inhibitor known as CA. Upon recognition and phosphorylation of Tyr‐Pep by over expressed MET in cells, it disrupts LLPS propensity and facilitates the disintegration of WntSI. Consequently,this enables it to suppress the carcinogenic effect mediated by β‐catenin,effectively overcoming acquired resistance to EGFR‐TKIs caused by MET amplification in both cell line‐derived and patient‐derived tumor xenograft (PDX) mouse models while maintaining exceptional biosecurity. This effective strategy not only suppresses the Wnt/β‐catenin signaling pathway selectively, but also serves as an innovative example for pro‐drug development through biologically responsive LLPS.

## Graphical abstract

An ingeniously crafted Wnt condensative prodrug called WntSI, capable of precisely targeting the Wnt/β‐catenin pathway, is developed through reversible supramolecular self‐assembly driven by liquid–liquid phase separation (LLPS). This process involves a MET/pH dual‐responsive peptide (Tyr‐Pep) and a potent Wnt inhibitor known as CA for overcoming drug resistance caused by activation of the Wnt/β‐catenin pathway by MET amplification.

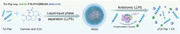

## 1 Introduction

The emergence of genomic profiling and subsequent targeted drug therapy has revolutionized the landscape of non‐small cell lung cancer (NSCLC) treatment in clinical settings, enabling patients with oncogene‐driven mutations, particularly EGFR mutation, to achieve remarkable response rates and documented improvements in progression‐free survival (PFS).^[^ ^1^, ^2^ ^]^ The development of resistance to these targeted therapies, however, is inevitable.^[^ ^3^ ^]^ Among them, the hepatocyte growth factor receptor (MET) amplification leads to acquired resistance to EGFR tyrosine kinase inhibitors (TKIs) in 15–22% of patients by activating proliferative signals of the ERBB3‐dependent PI3K pathway and subsequent downstream signaling pathways such as the MAPK and/or Wnt pathways.^[^ ^4^, ^5^, ^6^ ^]^ To address this challenge, the simultaneous inhibition of both EGFR and MET represents a theoretically viable therapeutic approach.^[^ ^7^ ^]^ The coadministration of MET inhibitors with Osimertinib (a third‐generation EGFR‐TKI), Gefitinib (a first‐generation EGFR‐TKI), or Erlotinib (a first‐generation EGFR‐TKI) has been supported by multiple preclinical and clinical studies as a potential strategy to prolong overall survival (OS) in patients with EGFR‐TKI‐resistant NSCLC harboring MET amplification.^[^ ^7^, ^8^, ^9^ ^]^ However, this strategy failed to exhibit the desired efficacy; on one hand, it only bestowed benefits upon a minority of patients with an overall response rate (ORR) below 15%, and on the other hand, even with treatment response, the prolonged OS consistently remained less than 6 months.^[^ ^7^, ^10^, ^11^ ^]^ Therefore, revealing the underlying mechanism of MET‐mediated resistance in oncogene‐driven NSCLC, particularly elucidating why the combined inhibition strategy targeting EGFR and MET exhibits insufficient efficacy, holds immense significance in devising novel and more efficacious treatment strategies to overcome this drug resistance.

To achieve this objective, two MET‐mediated acquired resistance NSCLC cell lines with EGFR‐activating mutations were established through lentivirus stable transfection herein, resulting in the overexpression of MET in PC‐9 cells (EGFR exon 19 deletion, PC9 OE) and NCI‐H1975 cells (EGFR T790M mutation, NCI‐H1975 OE). Both PC‐9 OE and NCI‐H1975 OE cells exhibited resistance to Gefitinib (the first‐generation EGFR‐TKI) and Osimertinib (the third‐generation EGFR‐TKI), respectively, which could not be significantly alleviated by the MET inhibitor. The subsequent bioinformatics and biochemistry research revealed here that cells overexpressing MET exhibited abnormal activation of the Wnt/β‐catenin signaling pathway, which displayed a positive feedback loop and remained unresponsive to inhibition by a MET inhibitor. Furthermore, clinical bioinformatics data on EGFR‐mutant NSCLC patients with MET amplification further supported this hyperactivation of the Wnt/β‐catenin pathway. Therefore, aberrant activation of the Wnt/β‐catenin signaling pathway may be the Achilles' Heel of acquired resistance to EGFR‐TKIs resulting from MET amplification.

Nevertheless, the clinical application of Wnt inhibitors has not been approved by FDA due to their dose‐limiting toxicities, which can result in severe side effects such as myelosuppression, leukopenia, myocardial damage, and rhabdomyolysis.^[^ ^12^ ^]^ Given the crucial role of the Wnt/β‐catenin signaling pathway in stem cell differentiation and cellular homeostasis in healthy tissue, untargeted inhibition would inevitably lead to detrimental consequences.^[^ ^13^, ^14^, ^15^ ^]^ Therefore, in order to overcome this predicament and precisely target the vulnerable point of acquired resistance to EGFR‐TKIs caused by MET amplification, a meticulously designed Wnt condensative prodrug (WntSI) was synthesized in this study through reversible supramolecular self‐assembly and liquid–liquid phase separation (LLPS), involving a specifically engineered MET/pH‐dual responsive peptide (Tyr‐Pep) and a potent Wnt inhibitor known as carnosic acid (CA). As part of our design, in response to the acidic microenvironment of tumors, WntSI exhibits a charge‐activated caveolae‐mediated endocytosis by tumor cells. Subsequently, Tyr‐Pep within WntSI can be recognized and phosphorylated by MET, leading to the loss of its LLPS tendency with CA and facilitating the disintegration of WntSI within MET‐overexpressed cells. The incorporation of these two design features ensures that WntSI exclusively fulfills its role in suppressing Wnt signaling in MET‐overexpressed tumor cells, thereby enhancing its potential for clinical translation. More significantly, WntSI effectively overcame acquired resistance to EGFR‐TKIs resulting from MET amplification in both cell line‐derived and patient‐derived tumor xenograft mouse models, while demonstrating exceptional biosecurity. In conclusion, this viable strategy reported here not only exhibits remarkable selectivity in suppressing the Wnt/β‐catenin signaling pathway in response to both the acidic microenvironment and MET amplification of the tumor, but also serves as an exemplary demonstration of precision‐medicine‐guided target discovery and drug development.

## 2 Results

### 2.1 The Hyperactivation of the Wnt/β‐Catenin Pathway is a Prominent Characteristic in Acquired Resistance to EGFR‐TKIs Caused by MET Amplification

**Figure 1:**
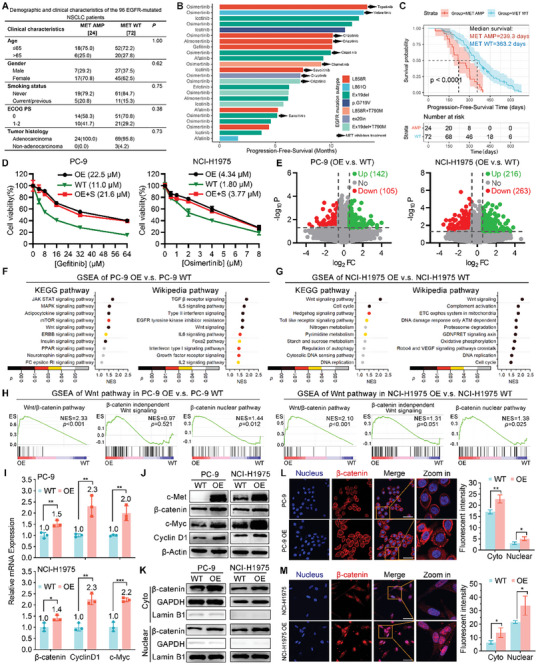
MET overexpression activates Wnt/β‐catenin signaling pathway and remotes nuclear translocation of β‐catenin. A) Baseline characteristics of 96 EGFR‐mutated non‐small cell lung cancer (NSCLC) patients. B) Swimmer plot of 24 patients with MET amplification (AMP) after treated with EGFR‐TKIs treatment. C) Kaplan‐Meier curves of progression‐free survival of MET AMP and MET WT NSCLC patients with EGFR‐mutation. *P*‐value is calculated by two‐sided log rank test. D) Cell viability of PC‐9/NCI‐H1975 WT and OE cells after exposure to different concentration of Gefitinib or Osimertinib (n = 3). E) Volcano plot of differentially expressed genes between PC‐9/NCI‐H1975 WT and OE cells. F,G) Bubble charts displaying the significantly altered Kyoto Encyclopedia of Genes and Genomes (KEGG) and Wikipedia pathways in Gene Set Enrichment Analysis (GSEA). Wnt signaling was significantly enriched in PC‐9/NCI‐H1975 OE cells compared to WT. The color of each bubble represents the significance of the pathway. H) GSEA showing the Wnt/β‐catenin pathway, β‐catenin independent Wnt signaling, and β‐catenin nuclear pathway differentially expressed in PC‐9/NCI‐H1975 WT and OE cells (n = 3). I)The relative mRNA expression of β‐catenin, Cyclin D1 and c‐Myc in PC‐9/ NCI‐H1975 WT and OE cells (n = 3). J) Western blotting analysis for the expression of c‐Met, β‐catenin, c‐Myc and Cyclin D1 protein levels in PC‐9/ NCI‐H1975 WT and OE cells. K) Cytoplasmic (cyto) and nuclear expression of β‐catenin in PC‐9/ NCI‐H1975 WT and OE cells were determined by Western blotting. GAPDH was used as reference protein for cytoplasmic protein. Laminin B1 was used as reference protein for nuclear protein. L,M) Localization and mean fluorescent intensity of cyto and nuclear of β‐catenin after MET OE by immunofluorescence (IF) assays in PC‐9 and NCI‐H1975 cells (n = 3). The data were presented as mean ± s.d. and comparisons were performed with Student's *t*‐test; ^*^ *p* < 0.05; ^**^ *p* < 0.01; ^***^ *p* < 0.001.

To investigate the urgency of MET amplification, a meticulous analysis was conducted on a total of 96 medical records from EGFR‐mutated NSCLC patients who developed acquired resistance to EGFR‐TKIs. All these patients had their paired plasma samples meticulously analyzed by NGS at disease progression and/or treatment discontinuation to identify the resistance mechanisms. Among them, 24 cases exhibited MET amplification (MET AMP) while the remaining 72 (MET WT) individuals experienced alternative mechanisms of drug resistance (**Figure** **1**A). The age, gender, smoking status, ECOG PS, and tumor histology exhibited no statistically significant differences between the two groups (Figure 1A), thereby affirming the rationality and randomness of the data. The subset analysis of EGFR mutation revealed that among the 24 cases with MET amplification (MET AMP), there appeared to be no discernible correlation between the duration of treatment with EGFR‐TKIs and the presence of EGFR mutation, while the MET inhibitor also failed to demonstrate any significant association with the PFS (Figure 1B). However, in comparison to the MET wild‐type group, the median progression‐free survival time in the MET amplification group exhibited a reduction of 123.9 days (from 363.2 days to 239.3 days), thereby indicating that MET amplification serves as an independent risk factor for the survival of patients with EGFR‐mutated NSCLC.

To further investigate the underlying mechanism of acquired resistance to EGFR‐TKIs caused by MET amplification, lentivirus‐mediated stable transfection was employed to generate two NSCLC cell lines overexpressing MET. One cell line, PC‐9, harbored the 19del EGFR mutation, while the other cell line, NCI‐H1975, possessed the EGFR T790M mutation. The presence of MET amplification was confirmed through RT‐qPCR analysis (Figure S1A, Supporting Information) and demonstrated a more active cell cycle (Figure S1B, Supporting Information). Moreover, MET overexpression significantly attenuated Gefitinib susceptibility in PC‐9 cells and diminished osimertinib susceptibility in NCI‐H1975 cells (Figure 1D). The subsequent step involved conducting RNA‐seq analysis to perform matched‐pair analysis on the MET amplification cell line (PC‐9 OE/NCI‐H1975 OE) and their corresponding wild type control (PC‐9 WT/NCI‐H1975 WT), which were treated with empty lentivirus. The amplification of MET, as depicted in Figure 1E, led to the up‐regulation of 142 genes in PC‐9 cells and 216 genes in NCI‐H1975 cells. Furthermore, the Gene Set Enrichment Analysis (GSEA) of tumor‐related signaling pathways in KEGG and Wikipedia revealed that the Wnt signaling pathway emerged as the predominant one capable of being enriched in both PC‐9 (Figure 1F) and NCI‐H1975 (Figure 1G) cell lines. Moreover, further GSEA analysis revealed that MET amplification activated the Wnt/β‐catenin signaling pathway (Figure 1H; Figure S1C, Supporting Information) rather than the β‐catenin‐independent Wnt signaling pathway (Figure 1H). This discovery was further supported by RT‐qPCR analysis (Figure 1I) and western blotting (WB) analysis (Figure 1J; Figure S1D, Supporting Information), which demonstrated a significant up‐regulation in the expression of β‐catenin and downstream proteins CyclinD1 and c‐Myc upon MET amplification. For further exploration of the underlying mechanisms, we delved into the subcellular localization of β‐catenin and made a noteworthy discovery: MET amplification significantly induced the accumulation of β‐catenin in both the nucleus and cytoplasm (Figure 1K–M; Figure S1E, Supporting Information). This finding aligns with previous reports suggesting that MET can phosphorylate Y142 on β‐catenin, thereby facilitating its translocation to the nucleus for Wnt‐mediated actions.^[^ ^16^, ^17^ ^]^

### 2.2 The Inhibition of MET Does Not Effectively Suppress the Wnt/β‐Catenin Signaling Pathway in Acquired Resistance to EGFR‐TKIs Caused by MET Amplification

**Figure 2:**
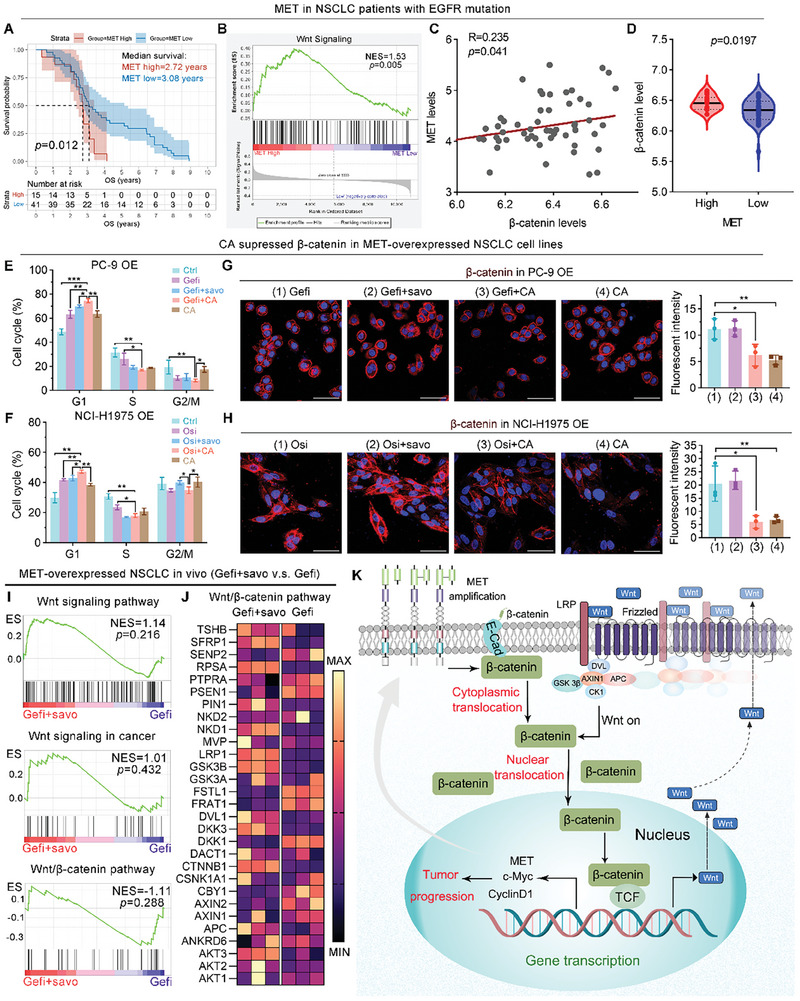
Wnt inhibitor carnosic acid (CA) can reduce the aberrant activation of Wnt/β‐catenin signaling pathway with positive feedback capacity induced by MET overexpression. A–D) Patients with EGFR mutations in public databases were integrated and divided into MET‐High and MET‐Low based on the optimal cut off value. Kaplan–Meier curves of overall survival of MET‐High and MET‐Low (A). *P*‐value is calculated by two‐sided log rank test. Gene Set Enrichment Analysis (GSEA) showing the Wnt signaling was more enriched in MET‐High patients (B), and a positive correlation between MET and β‐catenin levels was observed (C,D). Correlation analysis was conducted using the Spearman test. E,F) Cell cycles of PC‐9 OE and NCI‐H1975 OE cells were analyzed by flow cytometry (FCM) (n = 3). G,H) Expression and mean fluorescent intensity of β‐catenin after the indicated treatments by immunofluorescence (IF) assays in PC‐9 OE and NCI‐H1975 OE cells (scale bar: 50 µm, n = 3). I,J) GSEA and hierarchical clustering of genes results showing no statistically significant difference in Wnt signaling pathway, Wnt signaling in cancer, and Wnt/β‐catenin pathway in Gefitinib (Gefi) and Gefi combined with Savolitinib (Savo) in PC‐9 OE cell xenograft mouse model (n = 3). K) Mechanism diagram of aberrant activation of the Wnt/β‐catenin signaling pathway caused by MET OE. The data were presented as mean ± s.d. and comparisons were performed with Student's *t*‐test; ^*^ *p* < 0.05; ^**^ *p* < 0.01; ^***^ *p* < 0.001.

The crosstalk between MET amplification and the Wnt/β‐catenin signaling pathway was further investigated through gene expression and survival analysis in a cohort of 66 EGFR mutated NSCLC patients without any non‐tumor related diseases, which were carefully selected from the GEO database. The high expression of MET, as anticipated, significantly reduced the overall survival of patients with EGFR mutated NSCLC (**Figure** **2**A), while this subgroup with elevated MET expression exhibited an enrichment of up‐regulated Wnt signaling (Figure 2B). Furthermore, within this cohort of 66 EGFR mutated NSCLC patients, the expression of β‐catenin exhibited a positive correlation with MET expression (Figure 2C), and samples with elevated MET expression displayed heightened levels of β‐catenin (Figure 2D). In addition, we examined the expression of c‐Met and β‐catenin by immunohistochemical (IHC) staining in EGFR‐TKIs resistant NSCLC patients, and consistent with the results observed in the database, elevated levels of β‐catenin expression were detected in c‐Met overexpressing patients (Figure S2, Supporting Information). These findings suggest that inhibition of MET may effectively suppress the Wnt/β‐catenin signaling pathway in this particular case. The combination therapy of EGFR TKIs with the MET‐specific inhibitor Savolitinib exhibited enhanced cell cycle inhibition compared to monotherapy with EGFR TKIs in both PC‐9 OE and NCI‐H1975 OE cell lines (Figure 2E,F), presumably due to the supplementary suppression of c‐Met. Interestingly, Savolitinib demonstrated minimal inhibitory effects on both the protein levels of activated and total β‐catenin (Figure S3A, Supporting Information) as well as its nuclear translocation (Figure S3B–D, Supporting Information) when co‐treated with EGFR‐TKIs, contrasting sharply with the significant inhibition observed from carnosic acid (CA), a specific inhibitor targeting β‐catenin. Furthermore, the immunofluorescence images of β‐catenin once again provided support for this finding (Figure 2G,H). Importantly, these results collectively demonstrate that the inhibition of MET may not effectively suppress the Wnt/β‐catenin signaling pathway in acquired resistance to EGFR‐TKIs caused by MET amplification.

To further validate this in vivo, a nude‐mouse xenograft model of PC‐9 OE cells was established and subjected to treatment with Gefitinib or the combination of Gefitinib and Savolitinib. Consistent with the in vitro findings, the addition of Savolitinib did not exhibit any inhibitory effects on the Wnt/β‐catenin signaling pathway, as evidenced by both RNA‐seq analysis (Figure 2I,J) and IHC staining results (Figure S3E,F, Supporting Information). Based on these findings, we propose the following model: MET amplification triggers the translocation of β‐catenin from the cytoplasm to the nucleus, subsequently activating the positive feedback loop of the Wnt/β‐catenin signaling pathway (Figure 2K). In this process, Wnt10a, a prime molecule in the canonical Wnt/β‐catenin signaling pathway, was observed to be significantly upregulated in PC‐9 OE and NCI‐H1975 OE cell lines (Figure S4, Supporting Information). This activation promotes tumor progression and confers resistance to EGFR‐TKIs (Figure 2K). Consequently, inhibiting MET alone does not effectively suppress the Wnt/β‐catenin signaling pathway in this case. However, direct inhibition of β‐catenin could be a promising approach to overcome acquired resistance to EGFR‐TKIs caused by MET amplification.

### 2.3 The Design and Characteristics of the Wnt Supramolecular Inhibitor, WntSI

**Figure 3:**
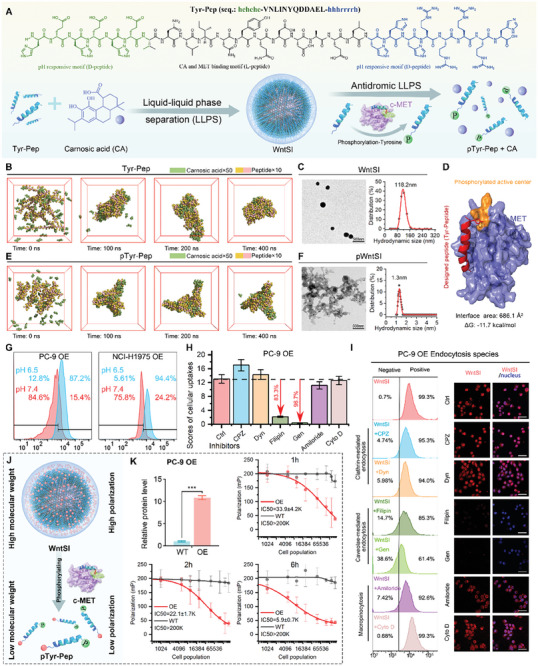
Physicochemical and pharmaceutical properties of WntSI. A) Schematic diagram of WntSI synthesis and disassembly mechanism. B,C) Simulation of Tyr‐Pep and carnosic acid (CA) molecular dynamics self‐assembly and corresponding transmission electron microscope (TEM) image and hydrodynamic diameter distribution. D) Binding site of MET protein to WntSI. E,F) Simulation of pTyr‐Pep and CA molecular dynamics self‐assembly and corresponding TEM image and hydrodynamic diameter distribution (The mass ratio of Tyr‐Pep/pTyr‐Pep and CA is 2:1). G) Uptakes of Cy5‐labeled WntSI by PC‐9 OE and NCI‐H1975 OE cells at pH 7.4 and pH 6.5 were analyzed by flow cytometry (FCM). H) Cellular uptake scores in PC‐9 OE cells after intervention with different endocytosis inhibitors (n = 3). I) FCM analysis results and laser scanning confocal microscope (LSCM) images of WntSI cellular uptake after intervention with different endocytosis inhibitors in PC‐9 OE cells (scale bar: 50 µm). J) WntSI is phosphorylated to pTyr‐Pep and CA by MET. K) Fluorescence polarization (FP) analysis of PC‐9 WT and OE cells after incubation with WntSI for 1, 2, and 6 hours (n = 3). The data were presented as mean ± s.d. and comparisons were performed with Student's *t*‐test; ^***^ *p* < 0.001.

However, the clinical implementation of Wnt inhibitors has not been sanctioned due to their dose‐limiting toxicities, which stem from the pivotal role played by the Wnt/β‐catenin signaling pathway in stem cell differentiation and cellular homeostasis within healthy tissue. Therefore, to surmount this predicament and precisely target the vulnerable point of acquired resistance to EGFR‐TKIs caused by MET amplification, a MET/pH‐dual responsive peptide (Tyr‐Pep) was ingeniously designed to achieve a reversible supramolecular self‐assembly and LLPS with the β‐catenin inhibitor CA. The Tyr‐Pep, in intricate detail, comprises three sequential segments from the N‐terminal to the C‐terminal: 1) an exquisite D‐enantiomer peptide motif of hehehe that exhibits a remarkable responsiveness to acidic pH conditions; 2) a captivating peptide motif VNLINYQDDAEL derived from β‐catenin, which not only interacts with CA but also undergoes phosphorylation by MET; and 3) an enchanting D‐enantiomer peptide motif of hhhrrrrh that demonstrates an extraordinary ability to respond to acidic pH and facilitate cell‐penetrating (**Figure** **3**A). The Tyr‐Pep, as per our design, is an intrinsically disordered region (IDR) that exhibits a propensity for LLPS. Furthermore, the binding of CA can augment their hydrophobicity, thereby further enhancing this LLPS tendency (Figure 3A).

The process was successfully replicated through a coarse‐grained dynamic simulation involving the interaction between Tyr‐Pep and CA molecules, wherein the emergence of LLPS and subsequent self‐assembly into a condensate were observed (Figure 3B). With the augmentation of the mass ratio of CA, the Tyr‐Pep/CA condensates exhibited a remarkable expansion in volume, as evidenced by the TEM image (Figure S5A, Supporting Information) and hydrodynamic size measured via dynamic light scattering (Figure S5B, Supporting Information), thereby substantiating the notion that CA amplifies this propensity for LLPS. Next, the mass ratio of 1:0.5 (Tyr‐Pep to CA) was selected for the preparation of the Wnt supramolecular inhibitor, WntSI, in order to maximize the utilization of condensate size for passive tumor targeting. This resulted in a hydrodynamic size of 118.2 nm (Figure 3C), which was conducive to promoting the enhanced permeability and retention (EPR) effect.^[^ ^18^, ^19^ ^]^ Furthermore, the successful assembly of WntSI was once again confirmed by both Fourier‐transform infrared (FT‐IR) spectrum analysis (Figure S5C, Supporting Information) and UV–vis absorption spectrum analysis (Figure S5D, Supporting Information), providing compelling evidence through the presence of characteristic absorption peaks associated with peptide and CA. More importantly, as our design WntSI was constructed by a reversible supramolecular self‐assembly driven by LLPS, which can disassemble in response to MET (Figure 3A). Alpha Fold 2.0 accurately predicted the strong bonding ability between Tyr‐Pep and c‐Met, as depicted in Figure 3D. This prediction was further substantiated through co‐immunoprecipitation analysis involving biotin‐labeled Tyr‐Pep and c‐Met, as illustrated in Figure S5E (Supporting Information). The findings demonstrated that Tyr‐Pep can be recognized by c‐Met, thereby showcasing its potential to undergo phosphorylation and transform into the more hydrophilic pTyr‐Pep, consequently reducing its inclination for LLPS (Figure 3E). Moreover, the TEM image and hydrodynamic size of c‐Met treated WntSI provided compelling evidence for the disassembly of this process into smaller‐sized molecules (Figure 3F). Besides, in order to provide evidence that the CA can be released during the phosphorylation process of Tyr‐Pep to pTyr‐Pep, a series of WntSIs with varying percentages of pTyr‐Pep were dissolved in PBS buffer. The concentration of CA in their supernatant after centrifugation at 15 000 g was measured using HPLC. As depicted in Figure S5F (Supporting Information), the dissociative CA increased proportionally with the percentage increase of pTyr‐Pep, reaching a 50% release point at 75% pTyr‐Pep. Furthermore, when all Tyr‐Pep was replaced by pTyr‐Pep, over 65% of CA could be released (Figure S5F, Supporting Information).

The WntSI design also incorporates a remarkable feature – its ability to respond to the acidic microenvironment (TME) of tumors, as demonstrated by the pH‐responsive charge reversal illustrated in Figure S6A (Supporting Information). The Cy5‐labeled WntSI was fabricated for further exploration by assembling CA with a Cy5‐labeled Tyr‐Pep peptide, which was synthesized through a spontaneous reaction between Cy5‐SE and the free amino group at the N‐terminal of Tyr‐Pep peptide. The characterization of the Cy5‐labeled Tyr‐Pep peptide was performed using LC‐MS (Figure S6B, Supporting Information). The D‐enantiomer peptide motif of hhhrrrrh, designed by us, exhibits this pH‐responsive behavior, leading to the conversion of the electroneutral imidazole moiety in histidine side chain into a positively charged form. This increased cationicity subsequently triggers the internalization of WntSI within cells. As anticipated, flow cytometry (FCM) results demonstrated that Cy5‐labeled WntSI exhibited a remarkable efficiency of cellular internalization, reaching ≈90%, in PC‐9 OE and NCI‐H1975 OE cells at pH 6.5. Conversely, the cell internalization efficiency was less than 25% at pH 7.4 (Figure 3G). Upon substitution of the pH‐responsive motif (hhhrrrrh) in Tyr‐Pep with its corresponding cationic D‐enantiomer peptide rrrrrrrr, the cationic WntSI displayed nearly identical potent cellular internalization capabilities at both pH 6.5 and pH 7.4 (Figure S6D, Supporting Information). These findings collectively indicate that the presence of the pH‐responsive motif confers controlled cellular internalization to WntSI upon acidification. Additionally, the Laser Scanning Confocal Microscope (LSCM) image of Cy5‐labeled WntSI revealed a notable disparity in its ability to penetrate T cells and macrophages at pH 7.4, as opposed to its efficient internalization into PC‐9 OE and NCI‐H1975 OE cells at pH 6.5 (Figure S6C, Supporting Information). To investigate the cellular internalization species of WntSI, a panel of six inhibitors representing diverse mechanisms of cell internalization were utilized: two inhibitors targeting clathrin‐mediated endocytosis, namely Chlorpromazine (CPZ) and Dynasore (DYN); two inhibitors inhibiting caveolae‐mediated endocytosis, Filipin and Genistein (GEN); as well as two inhibitors blocking macropinocytosis, Amiloride and Cytochalasin D (Cyto D). Interestingly, both Filipin and Genistein demonstrated remarkable efficacy not only in significantly inhibiting cellular internalization into PC‐9 OE (Figure 3H,I), but also in effectively suppressing it in NCI‐H1975 OE (Figure S6E,F, Supporting Information). These findings suggest that WntSI is internalized into tumor cells through a caveolae‐mediated endocytosis mechanism dependent on positive charge.

To confirm the ability of WntSI to respond to MET for disassembly post internalization, a fluorescence polarization (FP) method was employed to monitor the condensate state of RhB‐labeled WntSI. High molecular weight condensed matter exhibited high polarization while low molecular weight peptide monomer showed low polarization (Figure 3J). The FP of WntSI in both PC‐9 and NCI‐H1975 cells, as illustrated by Figure 3K and Figure S6G (Supporting Information), exhibited a decline dependent on the quantity of cells overexpressing MET, while remaining virtually unchanged in control cells. These findings demonstrate that WntSI disassembles in a manner contingent upon MET. Subsequently, to validate the intracellular disassembly of WntSI in response to the kinase region of MET intracellular domain, we incubated Cy5‐labeled Tyr‐pep‐conjugated WntSI with PC‐9 and NCI‐H1975 cell lines expressing MET, as well as MET‐knockdown cell lines. The knockdown of MET was confirmed by RT‐qPCR and WB analysis (Figure S7A,B, Supporting Information). As anticipated, the red fluorescence emitted from the Cy5‐labeled Tyr‐pep was dispersed throughout the cytoplasm in cells expressing MET, presenting a stark contrast to the concentrated bright spots observed in cells lacking functional MET (Figure S7C,D, Supporting Information). These findings strongly suggest that WntSI undergoes intracellular disassembly in a manner dependent on MET activity. Furthermore, to investigate the dissociation of CA from Tyr‐Pep, a double fluorescently labeled WntSI was synthesized by substituting 10% of CA with 5‐carboxyfluorescein, which is a fluorophore possessing a similar molecular structure to CA, and Cy5‐labeled Tyr‐pep. LSCM images revealed that the co‐localization of green FAM‐CA with red Tyr‐Pep gradually decreased after 6, 12, and 20 h of incubation in PC‐9 OE cells (Figure S8, Supporting Information), indicating a progressive intracellular disassembly of WntSI in MET‐overexpressed cells. Importantly, Cy5‐labeled Tyr‐Pep alone exhibited limited efficiency in entering PC‐9 OE cells (Figure S9, Supporting Information), further confirming the intracellular occurrence of WntSI disassembly.

### 2.4 The WntSI Effectively Inhibited the Wnt/β‐Catenin Signaling Pathway and Alleviated the Acquired Resistance to EGFR‐TKIs Caused by MET Amplification

**Figure 4:**
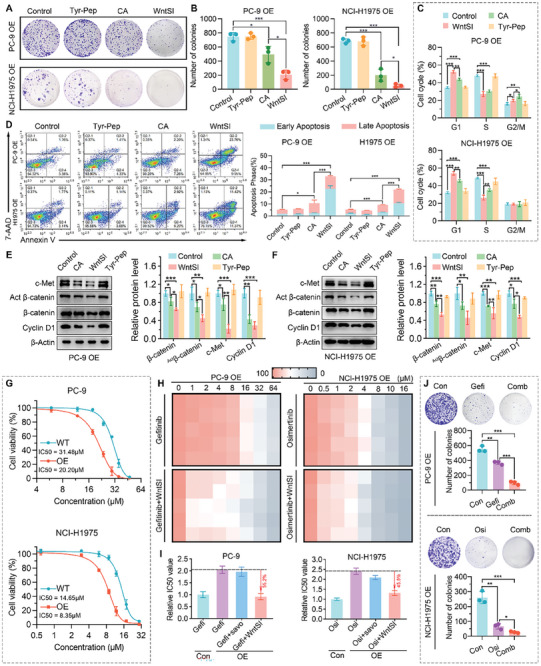
WntSI effectively inhibited the Wnt/β‐catenin pathway in MET overexpressed non‐small cell lung cancer cells in vitro. A,B) Colony formation assay and analysis of PC‐9 OE and NCI‐H1975 OE cells (n = 3). C) Cell cycles of PC‐9 OE and NCI‐H1975 OE cells were analyzed by flow cytometry (FCM) (n = 3). D) Apoptosis of PC‐9 OE and NCI‐H1975 OE cells were analyzed by FCM (n = 3). E,F) Western blotting analysis for the expression of c‐Met, active β‐catenin, β‐catenin, and Cyclin D1 protein levels in PC‐9 OE and NCI‐H1975 OE cells (n = 3). G) Dose‐response curves showing the response of WntSI to MET in PC‐9/NCI‐H1975 WT and OE cells. The results were measured by alamarBlue assay (n = 3). H) Cell viability heatmap of PC‐9 OE and NCI‐H1975 OE cells after treated with Gefitinib (Gefi) (PC‐9 OE), Gefi combined with WntSI (PC‐9 OE), Osimertinib (Osi) (NCI‐H1975 OE), or Osi combined with WntSI (NCI‐H1975 OE) (n = 5). I) Relative IC50 value in PC‐9/NCI‐H1975 control and OE cells after the indicated treatments (n = 3). J) Colony formation assay and analysis of PC‐9 OE and NCI‐H1975 OE cells after the indicated treatments (n = 3). The data were presented as mean ± s.d. and comparisons were performed with Student's *t*‐test; ^*^ *p* < 0.05; ^**^ *p* < 0.01; ^***^ *p* < 0.001; ns. Not significant.

To investigate the biofunction of WntSI, a comparative analysis was conducted on both the MET‐overexpressed cell line, PC‐9 OE, and NCI‐H1975 OE, involving WntSI, CA, and Tyr‐Pep. The suppressive effect of WntSI on clone formation surpassed that of CA, while Tyr‐Pep had no impact on either cell line (**Figure** **4**A,B). Moreover, WntSI effectively induced cell cycle arrest and apoptosis in a more pronounced manner compared to CA, while Tyr‐Pep also exhibited minimal impact on either cell line (Figure 4C,D). Furthermore, at the protein level, WntSI effectively suppressed the expression of c‐Met, active β‐catenin, total β‐catenin and its downstream protein CyclinD1 to a greater extent than that achieved by CA (Figure 4E,F). The aforementioned findings suggest that WntSI effectively impedes the Wnt/β‐catenin signaling pathway to a greater extent than CA, presumably due to the ingeniously crafted nanostructure of WntSI rather than the mere pharmacodynamic superposition derived from Tyr‐Pep. To validate the pharmacokinetics of WntSI, the fluorescence intensity of CY5‐SE labeled WntSI in the plasma of C57BL/6 mice at different time points was examined and quantified. WntSI exhibited a good blood long circulation with a half‐life of 10.25 ± 3.79 h (Figure S10, Supporting Information). Subsequently, we further investigated the organ distribution of WntSI in BALB/c nude mice. After injection of Cy5‐SE labeled WntSI for 2, 4, 6,12, and 24 h, the tumor tissues and major organs (heart, liver, spleen, lung, and kidney) were collected and their fluorescence signal intensities were determined using IVIS spectrum (Figure S11A, Supporting Information). From the distribution results and the statistics of tumor‐to‐organ fluorescence ratio, it is evident that WntSI can be highly enriched in tumor tissues with a time‐dependent tendency (Figure S11B, Supporting Information). Taken together, these results indicate that WntSI effectively inhibits the Wnt/β‐catenin pathway in vitro and has a satisfactory tumor‐targeting circulation time in vivo.

Besides, WntSI exhibited enhanced efficacy in suppressing cell viability in MET‐overexpressing cells compared to the control with low levels of MET expression (Figure 4G), thereby further supporting its cytotoxicity activated by MET. The WntSI is anticipated to effectively alleviate the acquired resistance induced by MET amplification to EGFR‐TKIs, thereby remarkably restoring the drug susceptibility of Gefitinib in PC‐9 OE cells and Osimertinib in NCI‐1975 OE cells (Figure 4H,I). In contrast, Savolitinib exhibited negligible impact on the susceptibility of EGFR‐TKIs (Figure 4H,I). Furthermore, the combined treatment of WntSI with Gefitinib or Osimertinib significantly reduced clone formation of PC‐9 OE and NCI‐1975OE to a greater extent than Gefitinib or Osimertinib monotherapy (Figure 4J). Recollectively, WntSI effectively mitigated the acquired resistance to EGFR‐TKIs induced by MET amplification.

### 2.5 WntSI Alleviated the Acquired Resistance to Gefitinib Caused by MET Amplification In Vivo

**Figure 5:**
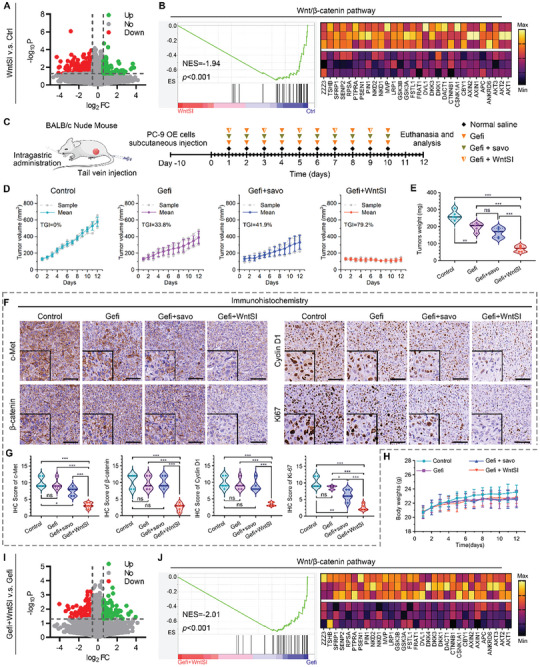
WntSI alleviated EGFR‐TKIs resistance in PC‐9 OE cell xenograft mouse model. A) Volcano plot of differentially expressed genes between control (ctrl) and WntSI group. B) Gene Set Enrichment Analysis (GSEA) and hierarchical clustering of genes results showing the Wnt/β‐catenin pathway differentially expressed in response to WntSI treatment (n = 3). C) Schematic diagram of the drug intervention protocol, mice in different groups were treated with control (normal saline), Gefitinib (Gefi), Gefi combined with Savolitinib (savo), and Gefi combined with WntSI (n = 5). D) Tumor growth curve of mice in each group (n = 5). E) Tumor weight of mice in each group after the indicated treatments (n = 5). F,G) Immunohistochemistry (IHC) and scores of c‐Met, β‐catenin, Cyclin D1, and Ki67 in representative tumor sections after the indicated treatments (scale bar: 100 µm, n = 5). H) Body weight of mice in each group during the indicated treatments (n = 5). I) Volcano plot of differentially expressed genes between Gefi and Gefi combined with WntSI group. J) GSEA and hierarchical clustering of genes results showing the Wnt/β‐catenin pathway differentially expressed in response to Gefi combined with WntSI compared to Gefi monotherapy (n = 3). The data were presented as mean ± s.d. and comparisons were performed with Student's *t*‐test; ^*^ *p* < 0.05; ^**^ *p* < 0.01; ^***^ *p* < 0.001; ns. Not significant.

To evaluate the inhibitory impact of WntSI on the Wnt/β‐catenin pathway in vivo, we established a model of PC‐9 OE cell xenografts in BALB/c nude mice (Figure S12A, Supporting Information). Photographs and weights of tumors as well as the recorded tumor volume curves showed that WntSI was more effective than CA in tumor growth inhibition (Figure S12B–D, Supporting Information). The IHC staining result further indicated that WntSI effectively inhibited the Wnt/β‐catenin pathway in vivo (Figure S12E, Supporting Information). In addition, we evaluated the drug safety of CA and WntSI, including recording the weight fluctuation of mice during the treatment period as well as the detection of hematological and biochemical indicators (Figure S12F–H, Supporting Information). The results showed that the weight growth rate of the CA group was slowed down, the levels of white blood cells (WBC), neutrophils and platelets were significantly lower than those of the control group, and the levels of aspartate transaminase (ALT) and alanine aminotransferase (AST) showed varying degrees of increase. Of note, this phenomenon was not observed in the WntSI group of mice, suggesting that the MET/pH dual target strategy of WntSI greatly reduced the side effects of Wnt inhibitors. The RNA‐seq analysis and subsequent GSEA results (**Figure** **5**A,B) provide compelling evidence that the Wnt/β‐catenin pathway is significantly down‐regulated in the WntSI intervention group compared to the mock‐treated group. To further evaluate the potential of WntSI in combination with EGFR‐TKIs to overcome resistance caused by MET overexpression/amplification, we divided nude mice bearing PC‐9 OE xenograft tumors into four groups: control (normal saline), Gefitinib (25 mg kg^−1^ d^−1^), Gefitinib combined with Savolitinib (2.5 mg kg^−1^ d^−1^) and Gefitinib combined with WntSI (3 mg kg^−1^, every two days) group. The specific treatment regimen is illustrated in Figure 5C. After 12 days of initial treatment, we evaluated the tumor suppressive efficacy of Gefitinib alone, as well as in combination with Savolitinib or WntSI. Remarkably, the combination of Gefitinib and WntSI exhibited a significantly superior inhibition of tumor growth compared to other groups, irrespective of tumor volume or weight (Figure 5D,E). Conversely, the co‐administration of Gefitinib and Savolitinib did not effectively impede tumor growth (tumor growth inhibition (TGI) value: Gefitinib vs Gefitinib combined with Savolitinib, 33.8% vs 41.9%). Furthermore, we conducted IHC staining and IHC scoring (Figure 5F,G) to explore the expression of c‐Met, β‐catenin pathway‐associated proteins, as well as Ki67 in the tumor tissues. Remarkably, the combination of Gefitinib with WntSI exhibited a significant reduction in the levels of c‐Met, β‐catenin, Cyclin D1, and Ki67. Notably though, neither Gefitinib alone nor its combination with Savolitinib showed any decrease in the levels of β‐catenin and Cyclin D1 compared to the control group (Figure 5F,G). The RNA‐seq analysis and subsequent GSEA results (Figure 5I,J) further bolstered the aforementioned conclusions, providing additional validation for the efficacy of combining Gefitinib with WntSI in EGFR mutant lung cancer tumors exhibiting MET overexpression/amplification. This synergistic approach serves as a potent antitumor strategy to overcome resistance to EGFR‐TKIs caused by MET overexpression/amplification. In addition, we closely monitored the fluctuations in body weight among the four groups of mice throughout the course of treatment (Figure 5H). Furthermore, we meticulously examined the hematological (Figure S13A, Supporting Information) and biochemical (Figure S13B, Supporting Information) indicators of the mice post‐treatment, alongside conducting hematoxylin‐eosin (H&E) staining on their vital organs (Figure S13C, Supporting Information), all revealing an absence of any treatment‐related toxic events.

### 2.6 The Biosafety Evaluation of WntSI on Healthy C57BL/6 Mice

**Figure 6:**
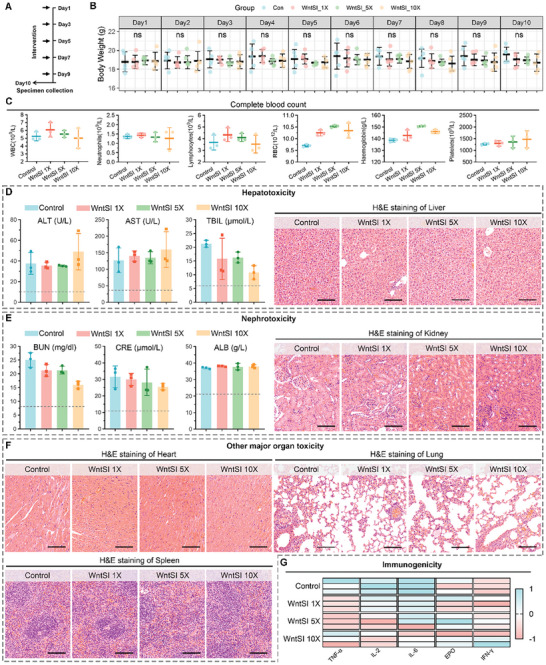
Evaluation of the biosafety of WntSI on healthy C57BL/6 mice. A) Schematic diagram of in vivo safety evaluation of WntSI. B) Body weight changes in mice during control group, WntSI 1× (3 mg kg^−1^), 5× (15 mg kg^−1^), and 10× (30 mg kg^−1^) dose intervention groups (n = 5). C) White blood cell (WBC), neutrophils, lymphocyte, red blood cell (RBC), hemoglobin and platelets were examined in mice blood after indicated treatments (n = 3). D) Hepatotoxicity of WntSI was detected by aspartate transaminase (ALT), alanine aminotransferase (AST), total bilirubin (TBIL) and hematoxylin‐eosin (H&E) staining of liver (scale bar: 100 µm, n = 3). E) Nephrotoxicity of WntSI was detected by blood urea nitrogen (BUN), creatinine (CRE), albumin (ALB) and H&E staining of kidney (scale bar: 100 µm, n = 3). F) Cardiotoxicity, pulmonary toxicity and splenic toxicity of WntSI were detected by H&E staining (scale bar: 100 µm). G) Immunogenicity of WntSI in immunocompetent C57BL/6 mice was determined by the levels of tumor necrosis factor‐α (TNF‐α), interleukin‐2 (IL‐2), interleukin‐6 (IL‐6), erythropoietin (EPO), and interferon γ (IFN‐γ) (n = 3). The data were presented as mean ± s.d. and comparisons were performed with Student's *t*‐test; ns. Not significant.

Although numerous ongoing clinical trials are focused on Wnt/β‐catenin therapy, none of these medications have currently received clinical approval due to the occurrence of severe side effects resulting from their dose‐limiting toxicity.^[^ ^20^, ^21^ ^]^ For further clinical translation, we conducted a safety assessment of dose accumulation for WntSI in healthy C57BL/6 mice (**Figure** **6**A). We established three groups with different concentrations of WntSI: 1× (3 mg kg^−1^), 5× (15 mg kg^−1^), and 10× (30 mg kg^−1^). The control group was administered with PBS. WntSI was injected into the tail vein every two days for a total of five administrations. In the 10‐day administration model, there were no significant changes in body weight observed among mice in the WntSI 1×, 5×, and 10× dose concentration groups compared to the control group (Figure 6B). Furthermore, post‐treatment haematological tests did not reveal any adverse effects such as haemolysis, myelosuppression, anaemia, or thrombocytopenia (Figure 6C; Figure S14, Supporting Information). Hepatotoxicity and nephrotoxicity were assessed through biochemical indicators, including ALT, AST, total bilirubin (TBIL), blood urea nitrogen (BUN), creatinine (CRE), albumin (ALB) and H&E staining of pathological sections (Figure 6D,E). Notably, no abnormal morphological or biochemical changes were observed in any of the four groups. Furthermore, there were no pathological morphological changes detected in the hearts, lungs, or spleens of WntSI‐treated mice (Figure 6F). The immunogenicity of WntSI was assessed by testing inflammatory factors in mice serums, and the results revealed that even in the high‐dose intervention group, there was no significant increase observed in the levels of tumor necrosis factor‐α (TNF‐ α), interleukin‐2 (IL‐2), interleukin‐6 (IL‐6), erythropoietin (EPO), and interferon γ (IFN‐ γ) (Figure 6G). These findings collectively demonstrate the safe accumulation of doses for WntSI and its potential for clinical translation.

### 2.7 WntSI Alleviated the Acquired Resistance to Gefitinib in NSCLC Patient‐Derived Xenograft Model

**Figure 7:**
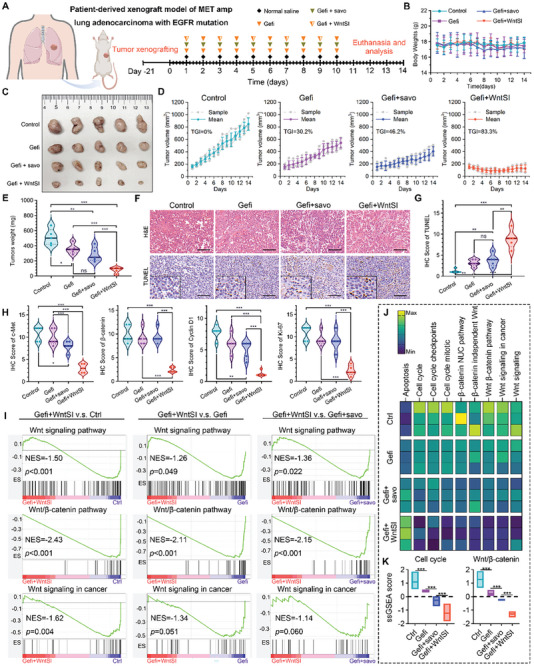
WntSI in vivo inhibited EGFR mutant with MET amplification NSCLC patient derived xenograft (PDX) model in BALB/c nude mice. A) Schematic diagram of PDX model construction and drug intervention protocol. B) Body weight of mice in each group during the indicated treatments (n = 5). C,D) Photographs of tumor and tumor growth curve of mice in each group (n = 5). E) Tumor weight of mice in each group after the indicated treatments (n = 5). F) Hematoxylin‐eosin (H&E) and TUNEL staining in representative tumor sections from mice with the indicated treatments (scale bar: 100 µm). G,H) Immunohistochemistry (IHC) scores of TUNEL, c‐Met, β‐catenin, Cyclin D1, and Ki67 in each group after the indicated treatments (n = 5). I) Gene Set Enrichment Analysis (GSEA) showing the Wnt signaling pathway, Wnt/β‐catenin pathway, and Wnt signaling in cancer differentially expressed in response to Gefitinib (Gefi) combined with WntSI compared to control, Gefi, Gefi combined with Savolitinib (savo) (n = 3). J,K) Heatmap of ssGSEA and ssGSEA score analysis in each group after the indicated treatments (n = 3). The data were presented as mean ± s.d. and comparisons were performed with Student's *t*‐test; ^*^ *p* < 0.05; ^**^ *p* < 0.01; ^***^ *p* < 0.001; ns. Not significant.

To further investigate the antitumor efficacy of WntSI in patients with NSCLC, we established a patient‐derived xenograft (PDX) model of lung adenocarcinoma harboring EGFR mutation and MET amplification in NOD/SCID mice (**Figure** **7**A). Drug interventions were administered after day 21 of subcutaneous inoculation, and the specific treatment regimen is depicted in Figure 7A. The dosage of administration remained consistent with the aforementioned regimen, during which the fluctuations in body weight among the four groups of mice were closely monitored, revealing no significant signs of weight loss within each group (Figure 7B). The tumor photographs (Figure 7C) vividly demonstrate the remarkable antitumor effects of Gefitinib combined with WntSI, as evidenced by the tumor volume curves and TGI value (Figure 7D). Furthermore, when compared to both the Gefitinib group and the combination of Gefitinib with Savolitinib groups, the tumor weight (Figure 7E) was significantly reduced in the presence of Gefitinib combined with WntSI. We conducted H&E, TUNEL, and IHC staining on the tumor tissues. As depicted in Figure 7F and Figure 7G, a substantial number of apoptotic cells were observed in the Gefitinib combined with WntSI group compared to the other groups. IHC staining and scoring demonstrated that WntSI significantly downregulated protein levels of c‐Met, β‐catenin, and Cyclin D1, while also leading to a significant decrease in the cell proliferation index Ki67 (Figure 7H; Figure S15A, Supporting Information). WB analysis of tumor tissue revealed effective suppression of Wnt/β‐catenin pathway‐related proteins in the Gefitinib combined with WntSI group (Figure S15B,C, Supporting Information), which was consistent with the GSEA results (Figure 7I). Additionally, the results of the heatmap analysis (Figure 7J) and statistical analysis (Figure 7K) based on ssGSEA further substantiated that the combination of Gefitinib with WntSI significantly impeded both the Wnt/β‐catenin pathway and cell cycle‐related pathways in comparison to other groups. Following treatment, morphological observations and blood chemistry tests were conducted, revealing no significant alterations among the groups (Figure S16A,B, Supporting Information). In summary, our findings suggest that WntSI effectively suppresses the Wnt/β‐catenin pathway in the PDX model, and when combined with Gefitinib, it can successfully reverse EGFR‐TKIs resistance induced by MET amplification.

## 3 Discussion

Both preclinical and clinical evidence strongly suggest that MET activation serves as a secondary driver for acquired resistance to EGFR‐TKIs therapy within specific genomic subsets.^[^ ^22^, ^23^ ^]^ To overcome this challenge, a combined treatment approach involving EGFR‐TKIs and MET‐TKIs has been extensively investigated as a promising strategy to provide therapeutic benefits for patients with EGFR‐mutant NSCLC who have experienced disease progression on previous EGFR‐TKIs, regardless of the presence of EGFR T790M mutation, attributed to MET amplification.^[^ ^8^, ^24^ ^]^ However, the ORR and the prolonged OS of the combined treatment is unsatisfactory,^[^ ^7^, ^10^, ^11^ ^]^ thus prompting a surge in recent years of research endeavors aimed at unraveling the intricate mechanisms underlying acquired resistance attributed to MET amplification.^[^ ^25^, ^26^ ^]^ The activation of the MET pathway in NSCLC is believed to occur through the activation of downstream RAS/ERK/MAPK, PI3K/AKT, and Wnt/β‐catenin signaling pathways that exert influence on properties governing cancer cell survival, proliferation, and invasiveness.^[^ ^7^, ^27^ ^]^ Among these MET downstream pathways, Wnt/β‐catenin is considered potential as an important mechanisms underlying acquired resistance attributed to MET amplification in patients with EGFR‐mutant NSCLC, because it has been found that HGF–MET pathway activation can induce Wnt/β‐catenin signaling and subsequently activates multidrug‐resistance‐associated protein‐1 expression and TKI resistance in tumor cells.^[^ ^24^, ^28^ ^]^ Furthermore, this work not only supports this perspective but also reveals that MET amplification triggers the translocation of β‐catenin into the nucleus. Subsequently, it activates the positive feedback loop of the Wnt/β‐catenin signaling pathway, thereby promoting tumor progression and conferring resistance to EGFR‐TKIs. Consequently, the inhibition of MET alone does not effectively suppress the Wnt/β‐catenin signaling pathway in this case, thereby providing a reasonable explanation as to why the combined treatment of EGFR‐TKIs and MET‐TKIs is unsatisfactory against acquired resistance attributed to MET amplification in patients with EGFR‐mutant NSCLC. More importantly, the present study has elucidated a potential solution to surmount this acquired resistance through direct suppression of the Wnt/β‐catenin signaling pathway.

However, the clinical implementation of Wnt inhibitors has not been authorized due to their dose‐limiting toxicities, which arise from the pivotal role played by the Wnt/β‐catenin signaling pathway in stem cell differentiation and cellular homeostasis within healthy tissue. To overcome the limitations imposed by dose‐related toxicity, we draw inspiration from the phenomenon of LLPS, which serves as a driving force for cells to form biomolecular condensates that regulate biomolecular function.^[^ ^29^, ^30^ ^]^ This concept inspires us to potentially encapsulate Wnt inhibitors within such condensates, enabling their controlled release upon MET activation. Moreover, several studies have demonstrated that peptide coacervates obtained through LLPS possess the ability to incorporate low‐molecular‐weight compounds and effectively traverse the cell membrane via an endocytosis‐independent pathway.^[^ ^31^, ^32^, ^33^ ^]^ Thus, the present study ingeniously devised a novel inhibitor, WntSI, by employing the reversible supramolecular self‐assembly driven by LLPS mechanism, which involved a meticulously designed MET/pH‐dual responsive peptide (Tyr‐Pep) and a potent Wnt inhibitor CA. In response to the acidic microenvironment of tumors, WntSI demonstrates a caveolae‐mediated endocytosis activated by its positive charge, thereby captivating tumor cells. The subsequent recognition and phosphorylation of Tyr‐Pep within WntSI by MET not only leads to the loss of its LLPS tendency with CA but also facilitates the disintegration of WntSI within MET‐overexpressed cells. The incorporation of these two design features, so to speak, ensures that WntSI exclusively fulfills its role in suppressing Wnt signaling in MET‐overexpressed tumor cells and significantly mitigates dose‐limiting toxicities, thereby amplifying its potential for clinical translation.

More significantly, WntSI effectively hinders the Wnt/β‐catenin signaling pathway to a greater extent than CA, owing to the ingeniously engineered nanostructure of WntSI rather than solely relying on pharmacodynamic superposition derived from Tyr‐Pep. Furthermore, WntSI proficiently alleviates acquired resistance caused by MET amplification in EGFR‐mutated NSCLC cell line against Gefitinib in PC‐9 cells and Osimertinib in NCI‐H1975 cells with T790M mutation. Most significantly, WntSI effectively overcame acquired resistance to EGFR‐TKIs resulting from MET amplification in both cell line‐derived and patient‐derived tumor xenograft mouse models, while exhibiting exceptional safety measures. In conclusion, the presented viable strategy not only demonstrates a clinical translational potential of a Wnt inhibitor with remarkable selectivity in suppressing the Wnt/β‐catenin signaling pathway in response to both the acidic microenvironment and MET amplification of the tumor, but also serves as an exemplary demonstration of precision medicine‐guided target discovery and drug development.

## 4 Experimental Section

### Patient and Datasets

The electronic medical records of patients were retrospectively analyzed with EGFR‐mutated lung cancer who were treated at the First Affiliated Hospital of Xi'an Jiaotong University. A total of 24 patients, who experienced disease progression after receiving EGFR‐TKIs and were confirmed to have MET amplification while testing negative for T790M acquired resistance mutation through next generation sequencing (NGS), were included in this study. Additionally, a 1:3 ratio was employed to include patients who experienced disease progression after treatment with EGFR‐TKIs but lacked MET amplification and T790M mutation as a comparator population (n = 72). Eligible patients were between the ages of 18 and 85 and presented with advanced NSCLC following clinical assessment. All patients underwent follow‐up, and PFS was defined as the duration from initiation of EGFR‐TKIs treatment to either disease progression or all‐cause mortality. The study received approval from the institution's Ethics Committee (XJTU1AF2023LSK‐399).

Moreover, two independent cohorts of lung cancer RNA expression data were obtained from the GEO database, specifically GSE11969 and GSE72094. The patients with EGFR mutation were identified based on their clinical information, and their clinical data including survival time, survival status, and EGFR mutation status were integrated. Subsequently, the expression profiles of patients with EGFR mutation from both datasets were extracted separately. The expression matrices of these two datasets were then merged using common genes. Finally, to mitigate batch effects, the ComBat function in the sva package was applied resulting in the establishment of an EGFR‐meta cohort consisting of individuals with EGFR mutations. The integrated cohort of patients was divided into MET‐high and MET‐low expression groups using a cut‐off value determined by the “surv_cutpoint” function from the “survminer” package in R software. Kaplan–Meier survival curves were constructed to compare OS between MET‐high and MET‐low groups, while also exploring the correlation between MET and β‐catenin.

### The Synthesis of Tyr‐Pep and pTyr‐Pep

Tyr‐Pep and pTyr‐Pep were synthesized using the solid‐phase polypeptide synthesis method based on Fmoc (N‐9‐fluorenylmethoxycarbonyl). The specific procedure is as follows: 520 mg of MBHA resin (loading: 0.48 mmol g^−1^) was weighed and added to a peptide synthesis reaction vessel, followed by swelling in 10 mL DMF/DCM (1:1, v/v) for 30 min. The solution was drained from the resin, and the Fmoc group was removed with 20% (v/v) piperidine in DMF for 5 min and then for an additional 10 min. After deprotection, Fmoc‐protected amino acids (5 equiv.), HBTU/HOBT (4.8 equiv.), and DIEA (5 equiv.) in DMF were added into the peptide synthesis reaction vessel for coupling for 30 min. The Fmoc deprotection step was carried out as described above. Following each coupling and Fmoc‐deprotection step, the peptide resins were washed with DMF (5 mL × 3), DCM (5 mL × 3), and DMF again (5 mL × 3). Finally, peptides were separated from the resin using a mixture of TFA (88%), H2O (5%), phenol (5%), and triisopropylsilane (TIPS) (2%) by volume, then precipitated in cold diethyl ether before being subjected to freeze drying on a lyophilizer. The crude product was isolated through semi‐preparative C18 HPLC purification and identified by ESI mass spectrometry.

### Fabrication, Characterization, and Physicochemical Analysis of WntSI

The as‐prepared 1 mg Tyr‐Pep was dissolved in 1 mL of PBS buffer (pH 7.4) and sonicated for 10 min. Subsequently, CA was dissolved in DMSO at a concentration of 0.1 mg µL^−1^, and then 5 µL of the CA stock solution was added dropwise into the PBS buffer containing Tyr‐Pep under ultrasonication. WntSI was obtained after sonication for 30 min and stored at 4 °C for further experiments.

The morphology of WntSI was observed using a transmission electron microscope (TEM) (Thermo Fisher Talos L120C G2) operated at 120 kV. Briefly, the prepared samples were dropped onto a copper mesh with different mass ratios of Tyr‐Pep to CA (1:4, 1:2, 1:1, and 1:0.5), allowed to sit for 30 min, and then the dried samples were examined by TEM to determine particle size. The hydrodynamic size distribution was obtained through dynamic light scattering (DLS) measurements using a Malvern Zetasizer Nano ZS system. The samples were diluted 50 times with double‐distilled water and sonicated for 15 min before being placed in the measuring cell and equilibrated for 120 s. The hydrodynamic diameter and number distribution of the various samples were determined based on three runs of the system. WntSI solutions with different pH values (pH 6.0, pH 6.5, pH 7.0, pH 7.4, pH8.0) were prepared to obtain zeta potential and zeta potential distribution data using laser Doppler microelectrophoresis on a Malvern Zetasizer Nano ZS system. The surface chemical structures of Tyr‐Pep, CA, and WntSI were evaluated using Fourier transform infrared spectroscopy (Nicolet6700). The UV–vis absorption spectrum from wavelengths ranging from 200 to 800 nm of Tyr‐Pep, CA, and WntSI were measured using a Shimadzu 3000 spectrophotometer for comparison purposes. As for computer simulations, the self‐assembly of Tyr‐Pep and pTyr‐Pep with CA was simulated using coarse‐grained molecular dynamics. Furthermore, molecular docking simulation was employed to predict the binding site of MET protein with Tyr‐Pep.

### Cell Cultures, RNA Interference and the Construction of MET Overexpression Cell Lines

PC‐9, NCI‐H1975, RAW 264.7, and Jurkat were obtained from the Cell Bank of the Chinese Academy of Sciences (Shanghai, China). PC‐9, NCI‐H1975, and Jurkat cell lines were cultured in RPMI 1640 medium, and RAW 264.7 cells were cultivated in DMEM medium. The MET siRNA synthesis was acquired from Tsingke (Beijing, China). The sequences were listed in Table S1 (Supporting Information). The MET OE lentivirus vector was commissioned to Genechem (Shanghai, China) for construction and packaging. 1 × 10^8^ transducing units (TU) of lentivirus were mixed with 40 µL of HitransG P (PC‐9) or HitransG A (NCI‐H1975) infection enhancement solution and 1 mL of complete medium to infect cells in a 6‐well plate. The specific steps are as follows: 500 µL of complete medium containing lentivirus and infection enhancement solution was added at a cell density of ≈15%, incubated for a total of 24 h, then the medium was changed and cultivation continued for another 48 h. Subsequently, complete medium containing puromycin at a concentration of 2.0 µg mL^−1^ was added for drug screening for 48 h.

### The Assessment of Cell Viability and Colony Formation Through In Vitro Assays

To evaluate the impact of Gefitinib intervention on the cell viability of PC‐9 and PC‐9 OE, as well as Osimertinib intervention on NCI‐H1975 and NCI‐H1975 OE, cells were treated with varying drug concentrations (Gefitinib: 0, 4, 8, 16, 32, and 64 µm; Osimertinib: 0, 0.5, 1, 2, 4, and 8 µm) for a duration of 48 h. In the presented cell experiments, Savolitinib was administered in combination with Gefitinib or Osimertinib at a concentration of 10 µm. The cell viability was determined by alamarBlue assay (Thermo Fisher Scientific).

The PC‐9 OE and NCI‐H1975 OE cells were seeded at a low density and cultured in serum‐rich medium containing drugs for 7–10 days to assess clone formation. Subsequently, the culture medium was discarded, and the cell colonies were fixed with methanol, stained with 0.1% crystal violet, and washed with distilled water until the background was clear. The number of colonies was quantified using Image J software.

### RT‐qPCR Assay

Total RNA was extracted using Trizol reagent (Ambion). Gene expression changes relative to GAPDH were calculated using the △△CT method. All primers are shown in Table S1 (Supporting Information).

### Western Blotting Analysis

The nuclear and cytoplasmic proteins were separated using the Nuclear and Cytoplasmic Protein Extraction Kit (Beyotime). The cells and tissue samples were subjected to specific treatments, followed by lysis in RIPA lysis buffer (Beyotime) supplemented with protease inhibitors (Beyotime). Primary antibodies against β‐catenin and c‐Myc were obtained from Abcam, while Cell Signaling Technology provided antibodies against Active β‐catenin, Cyclin D1, and c‐Met. Antibodies against β‐actin, Lamin B1, and GAPDH were sourced from Proteintech. Horseradish peroxidase‐conjugated secondary antibodies were obtained from Signalway Antibody.

### Biotin Affinity Purification Assay

The Biotin‐labeled Tyr‐Pep was pre‐incubated with streptavidin‐coupled magnetic beads (Epizyme) and subjected to three washes with binding buffers (50 mm Tris, 150 mm NaCl, 0.5% Triton X‐100, 0.5% Tween 20, pH7.5), followed by incubation with the lysed PC‐9 OE cells at 4 °C for 6 h. The protein bound to Tyr‐Pep was enriched through the high‐affinity interaction between biotin and streptavidin. Subsequently, the samples were washed again with binding buffer and utilized for subsequent western blotting analysis.

### The Fabrication of Cy5‐Labaled WntSI

The Cy5‐labeled WntSI was prepared by assembling CA with a Cy5‐labeled Tyr‐Pep peptide using the same preparation scheme as WntSI. The Cy5‐labeled Tyr‐Pep was synthesized through a spontaneous reaction between Cy5‐SE and the free amino group at the N‐terminal of Tyr‐Pep peptide. For this, 5 mg of Tyr‐Pep and 1 mg of Cy5‐SE were dissolved in PBS buffer at pH 8.0, with DMSO used to aid in dissolving the Cy5‐SE. After a 30 min reaction, the resulting Cy5‐labeled Tyr‐Pep could be purified by reverse HPLC and characterized by LC‐MS.

### The Cellular Internalization of WntSI

The cellular uptake of WntSI was assessed using a flow cytometer (BD Biosciences, NJ). PC‐9 OE and NCI‐H1975 OE cell lines were cultured in their respective growth medium for 24 h. Subsequently, the medium was replaced with conditioned medium containing Cy5‐labeled WntSI at pH 7.4 and pH 6.5, followed by further incubation at 37 °C for 6 h. FCM analysis was performed after washing the cells with PBS to remove excess Cy5‐labeled WntSI.

The PC‐9 OE, NCI‐H1975 OE, RAW 264.7, and Jurkat cells were cultured in confocal dishes for 24 h, followed by co‐incubation with Cy5‐labeled WntSI for 6 h. Subsequently, the cells were washed with PBS and fixed. After permeabilization with 0.2% Triton X‐100, the nuclei were stained with DAPI. Finally, a super‐resolution laser scanning confocal microscope (LSCM) (Olympus FV3000) was utilized to observe the cellular uptake.

To investigate the cellular uptake mechanism of WntSI in PC‐9 OE and NCI‐H1975 OE cells, serum‐free medium was pre‐incubated with six different endocytosis inhibitors (Chlorpromazine, Dynasore, Filipin, Genistein, Amiloride, and Cytochalasin D) for 1 h. Subsequently, Cy5‐labeled WntSI was added to continue incubation for 6 h. FCM and LSCM analysis were performed as previously described.

### The Technique of Fluorescence Polarization

The rhodamine (RhB)‐labeled WntSI was co‐incubated with PC‐9/NCI‐H1975 WT and OE cells for 1, 2, and 6 h, respectively. After centrifugation and resuspension of the cells in PBS, they were added to black opaque 96‐shallow well microplates. The fluorescence intensity was measured using a multi‐mode microplate reader (SpectraMax iD5). Background fluorescence was subtracted from the obtained data, and fluorescence polarization values were calculated separately. IC50 curves were generated using Origin software.

### The Transcriptome Analyzed using RNA‐Seq Technology

The cell samples underwent a 24 h drug intervention prior to total RNA extraction using Trizol (Ambion). The animal tumor tissues were promptly snap‐frozen in liquid nitrogen upon isolation from the body. Subsequently, the samples underwent mRNA fragmentation (300 bp), cDNA synthesis, library construction (NEBNext Ultra RNA Library Prep Kit for Illumina), PCR amplification, library quality control (Agilent 2100 Bioanalyzer), sequencing (Illumina HiSeq X Ten), and analysis.

### The Study Conducted on Mice

The mice used in this study were procured from the Laboratory Animal Center of Xi'an Jiaotong University. All experimental procedures involving animals strictly adhered to the guidelines set by the institution and received approval from the Laboratory Animal Center of Xi'an Jiaotong University (approval number: 2021‐1729).

### The PC‐9 OE NSCLC Cell Xenograft Mouse Models

5×10^6^ PC‐9 OE cells were subcutaneously injected into the right flanks of BALB/c nude mice aged 5–6 weeks. The tumor volume was calculated using the equation: volume = 1/2 × length × width^2^. Once the tumor volume reached 150–180 mm^3^, the mice were randomly grouped. The specific groups are as follows: 1) control (PBS), CA (3 mg kg^−1^), and WntSI (3 mg kg^−1^); the drug was administered via tail vein every two days for a total five injections; 2) control (normal saline), Gefitinib (25 mg kg^−1^ d^−1^), Gefitinib combined with Savolitinib (2.5 mg kg^−1^ d^−1^), and Gefitinib combined with WntSI (3 mg kg^−1^, every two days) groups; normal saline, Gefitinib, and Savolitinib were administered by daily gavage, while WntSI was administered via tail vein injection every two days. Tumor growth was monitored daily, and xenografts were collected for formalin‐fixed‐paraffin embedded (FFPE) or snap frozen in liquid nitrogen for subsequent experiments.

### The Patient‐Derived Xenografts (PDX) Model of NSCLC

The axillary transplantation of xenograft tissue from a patient with MET‐amplified and EGFR‐mutated NSCLC was conducted in NOD/SCID mice, followed by subsequent generations for further experimentation. Tumor specimens were sectioned into ≈5 mm tissue blocks and implanted into the axilla of BALB/c nude mice at 5 weeks old. After three weeks of inoculation, when the tumor volume reached ≈150 mm^3^, the mice were randomly assigned to control (normal saline), Gefitinib, Gefitinib combined with Savolitinib, and Gefitinib combined with WntSI groups. The intervention protocol remained consistent as before, and euthanasia was performed after 14 days of administration for collection of tumor tissue samples for further analysis.

### Biosafety Assessment

To evaluate the potential toxicity of WntSI, dose‐concentration groups were established at 1× (3 mg kg^−1^), 5× (15 mg kg^−1^), and 10× (30 mg kg^−1^). The control group was administered with PBS. C57BL/6 mice aged between 6–8 weeks were utilized for the experiments. Throughout the treatment period, the body weights of mice in each group were monitored, and on the 10th day after treatment, blood biochemistry indexes and major organ tissues were collected for toxicity assessment. Blood samples were obtained from the orbital region of mice. Hematological examination specimens were collected in anticoagulant tubes containing EDTAK2 while biochemical examination specimens were collected in eppendorf tubes. The latter specimens were left at room temperature for a duration of 2 h before being centrifuged at 2000 rpm for 15 min at a temperature of 4 °C; subsequently, they underwent another round of centrifugation to collect supernatant for testing purposes. Primary hematological indicators included RBC, HGB, WBC, NEU, LYMPH, and PLT whereas main biochemical indicators consisted of ALT, AST TBIL ALB BUN CRE levels. Major organs such as heart, liver, spleen, lung, and kidney underwent fixation using formalin neutral fixative (10%) followed by staining with hematoxylin‐eosin (H&E).

### ELISA Assay

The ELISA assay was performed by collecting blood from the orbital vein of mice and obtaining serum specimens after centrifugation. In order to assess the immunogenicity of WntSI, the levels of cytokines IL‐2, IL‐6, TNF‐α, IFN‐γ, and EPO were quantified. The serum was diluted fivefold using Specimen Universal Diluent and analyzed using ELISA kits (IL‐2, IL‐6, TNF‐α from NeoBioscience; EPO from mlbio) following the manufacturer's instructions. Three replicate wells were prepared for each group and the OD450 values of cytokines were measured for all groups.

### Statistical Analysis

The data were presented as mean ± standard deviation, and statistical analyses were conducted using two‐tailed Student's *t*‐test. Correlation analysis was conducted using the Spearman test. Sample size (n) for each statistical analysis was 3 or 5. Statistical analyses were performed using GraphPad Prism 8.0.1. A significance level of *P* < 0.05 was considered statistically significant.

## Conflict of Interest

The authors declare no conflict of interest.

## Supporting information

Supporting Information

## Data Availability

The data that support the findings of this study are available from the corresponding author upon reasonable request.
