# Supplementary material for: Reversing MET‐Mediated Resistance in Oncogene‐Driven NSCLC by MET‐Activated Wnt Condensative Prodrug
Source: Adv Sci (Weinh). 2024 Jun 13;11(30):2400603. doi: 10.1002/advs.202400603 (PMC11321677; doi:10.1002/advs.202400603)
Supplement: Supplementary file 1 — Supporting Information [file ADVS-11-2400603-s001.docx]

**Supplementary materials**

**Reversing MET-mediated Resistance in Oncogene-driven NSCLC by MET-activated Wnt Condensative Prodrug**

Na Liu^1,†^, Xiaoqiang Zheng^1,2,†^, Jin Yan^3,4,†^, Aimin Jiang^1^, Yu Yao^1^, Wangxiao He^1,2,5*^

1. Department of Medical Oncology, The First Affiliated Hospital of Xi'an Jiaotong University, Xi’an 710061, China.
2. Institute for Stem Cell & Regenerative Medicine, The Second Affiliated Hospital of Xi’an Jiaotong University, Xi’an 710004, China.
3. Department of infectious Diseases, The Second Affiliated Hospital of Xi'an Jiaotong University, Xi'an, 710004, PR. China
4. Department of Tumor and Immunology in precision medical institute, Western China Science and Technology Innovation Port, The Second Affiliated Hospital of Xi'an Jiaotong University, Xi'an, 710004, China.
5. Department of Talent Highland, The First Affiliated Hospital of Xi’an Jiao Tong University, Xi’an 710061, China.

* Corresponding authors:

Email: hewangxiao5366@xjtu.edu.cn (W. He)

**1. Supplementary Figures**


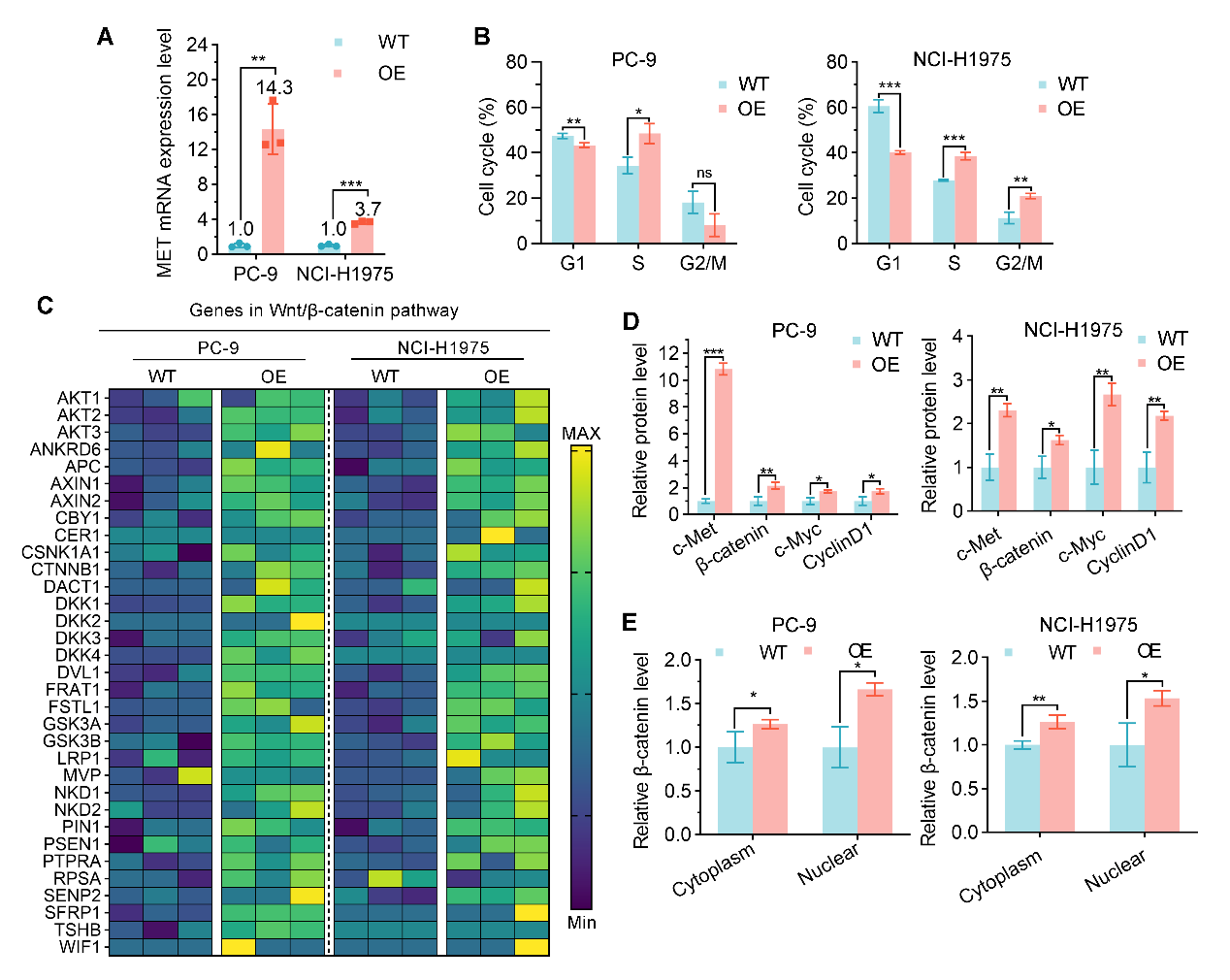


**Figure S1.** MET overexpression is associated with activation of the Wnt/β-catenin signaling pathway. A) Expression levels of MET mRNA in PC-9 and NCI-H1975 cells after transfection with MET overexpressed lentiviral vector (n = 3). B) Cell cycles of PC-9/NCI-H1975 WT and OE cells were analyzed by flow cytometry (n = 3). C) Heatmap of Wnt/β-catenin pathway gene changes in PC-9/NCI-H1975 WT and OE cells (n = 3). D) The protein levels of c-Met, β-catenin, c-Myc, and Cyclin D1 in PC-9 OE and NCI-H1975 OE cells (n = 3). E) The β-catenin protein levels of cytoplasm and nucleus in PC-9 OE and NCI-H1975 OE cells (n = 3). The data were presented as mean ± s.d. and comparisons were performed with Student’s t-test; *, *p* < 0.05; **, *p* < 0.01; ***, *p* < 0.001


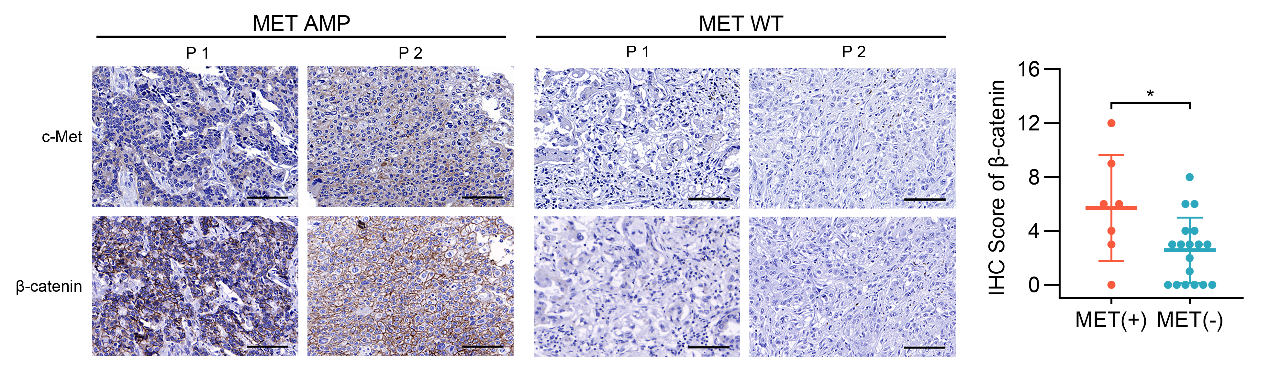


**Figure S2.** Representative IHC of c-Met and β-catenin for EGFR-TKI resistance patients with MET WT or AMP. scale bar, 100 μm**.** The data were presented as mean ± s.d. and comparisons were performed with Student’s t-test; *, *p* < 0.05


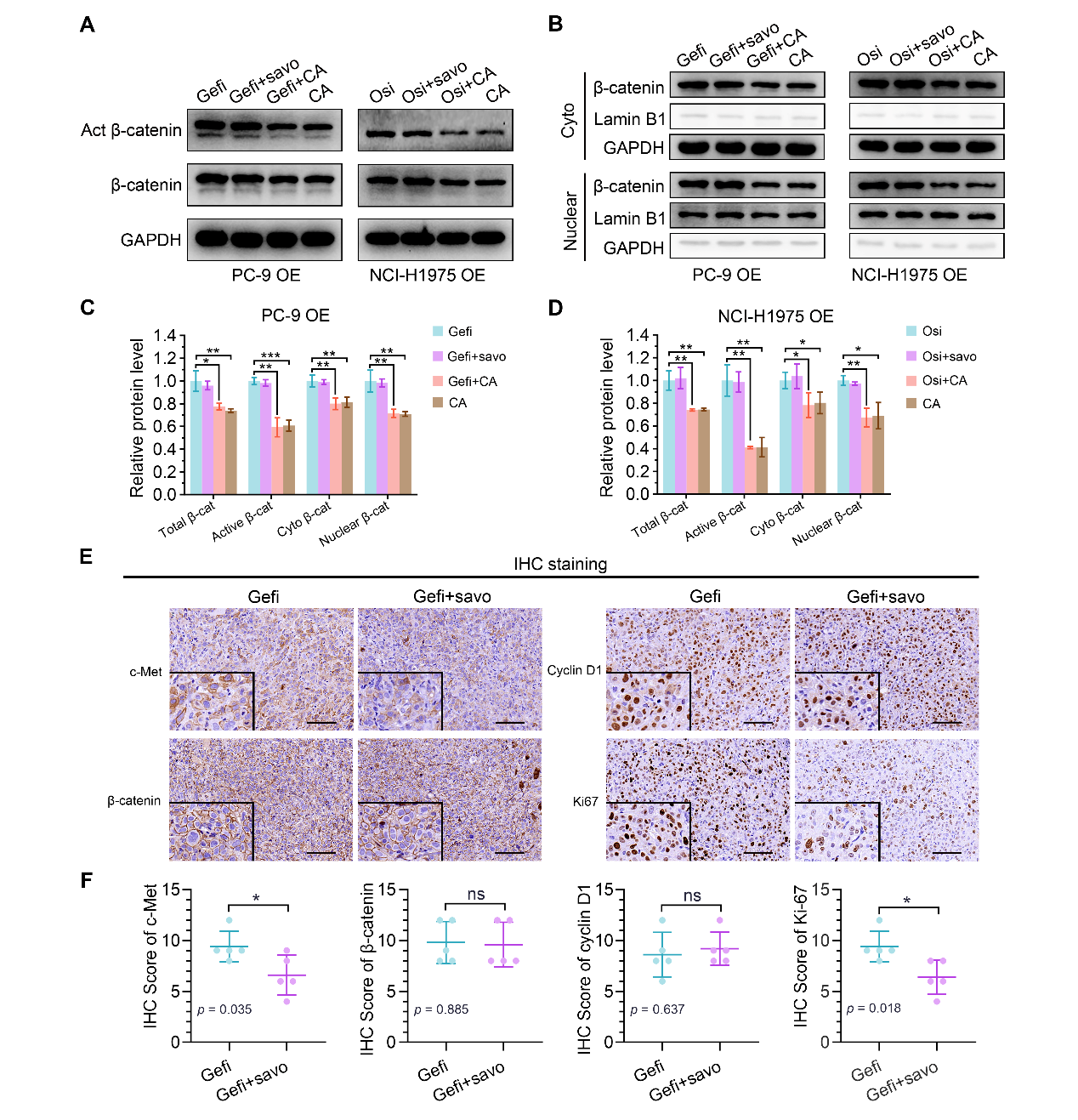


**Figure S3.** Activation of Wnt/β-catenin signaling pathway after MET overexpression cannot be inhibited by MET inhibitors. A) Western blotting analysis for the expression of active β-catenin and β-catenin protein levels in PC-9 OE and NCI-H1975 OE cells after indicated treatments. B) Cytoplasmic (cyto) and nuclear expression of β-catenin in PC-9 OE and NCI-H1975 OE cells were determined by western blotting. GAPDH was used as reference protein for cytoplasmic protein. Laminin B1 was used as reference protein for nuclear protein. C, D) The protein level of total β-catenin, active β-catenin, cyto β-catenin and nuclear β-catenin in PC-9 OE and NCI-H1975 OE cells after indicated treatments (n = 3). E, F) Immunohistochemistry (IHC) and scores of c-Met, β-catenin, Cyclin D1, and Ki67 in representative tumor sections after treatment with Gefitinib or Gefitinib combined with Savolitinib (scale bar: 100 μm, n = 5). The data were presented as mean ± s.d. and comparisons were performed with Student’s t-test; *, *p* < 0.05; **, *p* < 0.01; ns. Not significant.


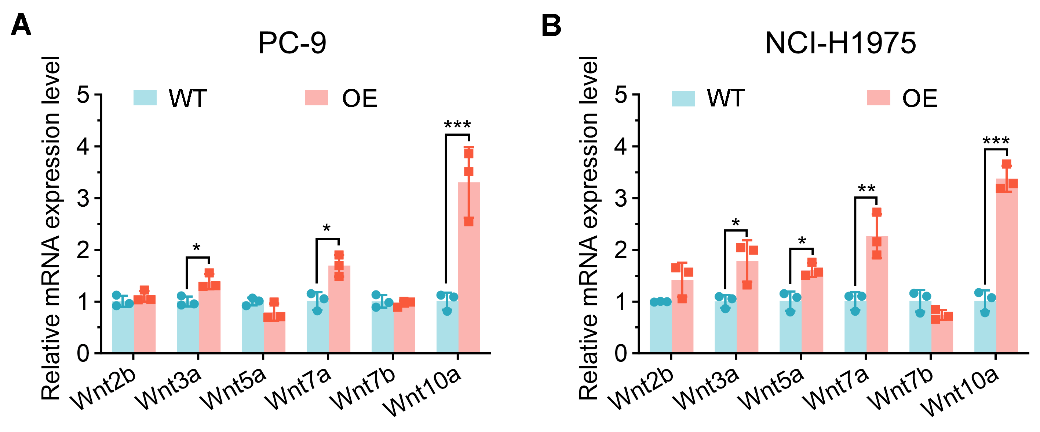


**Figure S4.** RT-qPCR revealed changes in the expression of main Wnt genes in MET overexpressing (OE) and wild type (WT) PC-9/NCI-H1975 cells (n = 3). The data were presented as mean ± s.d. and comparisons were performed with Student’s t-test; *, *p* < 0.05; **, *p* < 0.01; ***, *p* < 0.001


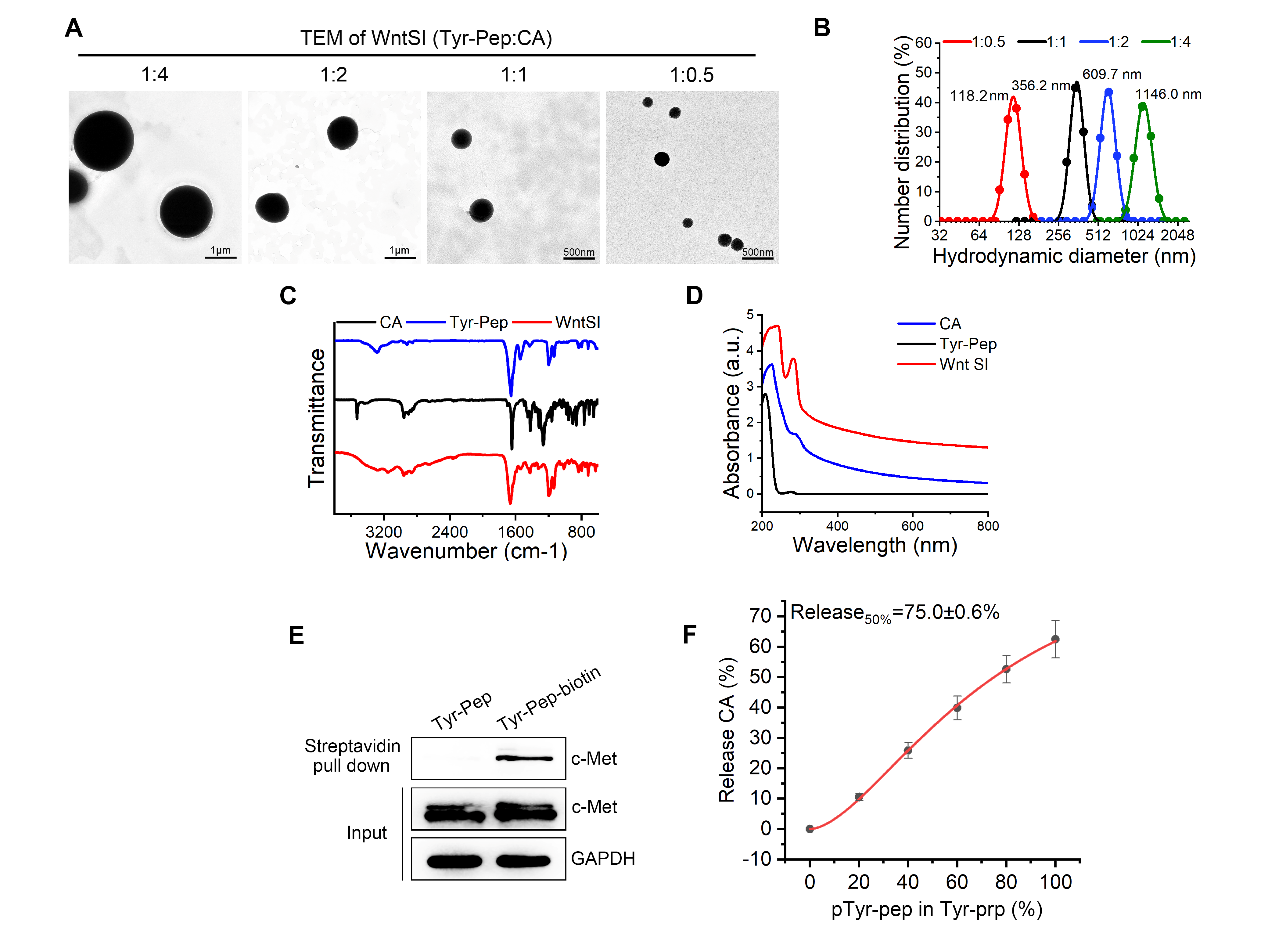


**Figure S5.** Characterization of WntSI. A, B) Transmission electron microscope (TEM) image and hydrodynamic diameter distribution of nanoclusters formed by liquid-liquid phase separation (LLPS) of Tyr-Pep induced by different concentrations of carnosic acid (CA). C, D) Fourier transform infrared spectroscopy (FT-IR) and ultraviolet-visible spectra of WntSI, CA, and Tyr-Pep. E) c-Met protein level in pull down assays using biotin-labeled Tyr-Pep conjugated to streptavidin magnetic beads. F) CA release curve of WntSI in response to the replacement of pTyr-Pep to Tyr-Pep (n = 3). The data were presented as mean ± s.d.


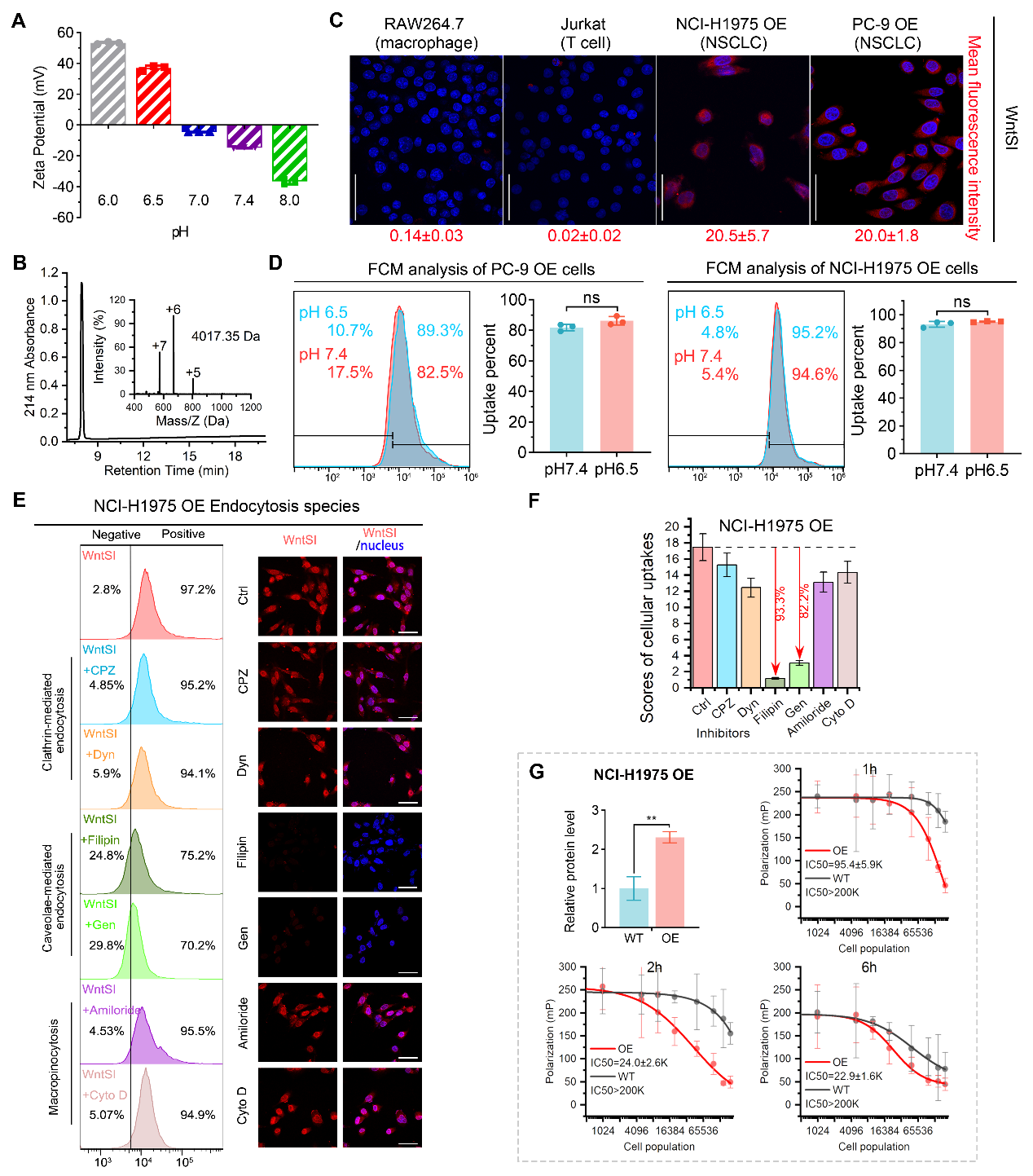


**Figure S6.** Physicochemical and pharmaceutical properties of WntSI. A) Zeta potential of WntSI with pH change was measured by dynamic light scattering (DLS) (n = 3). B) Cy5-labeled Tyr-Pep was detected by liquid chromatography-mass spectrometry (LC-MS). C) Laser scanning confocal microscopy (LSCM) showed the uptake of Cy5-labeled WntSI by PC-9 OE cells (pH 6.5), NCI-H1975 OE cells (pH 6.5), Jurkat (pH 7.4), and RAW 264.7 (pH 7.4) (scale bar: 50 μm). D) Flow cytometry (FCM) analysis of the uptake of WntSI (r8) by PC-9 OE and NCI-H1975 OE cells at pH 6.5 and pH 7.4 after replacement of pH responsive motif (hhhrrrrh) in Tyr-Pep by cell penetrating peptide (rrrrrrrr, r8). E) FCM analysis results and LSCM images of WntSI cellular uptake after intervention with different endocytosis inhibitors in NCI-H1975 OE cells (scale bar: 50 μm). F) Cellular uptake scores in NCI-H1975 OE after intervention with different endocytosis inhibitors (n = 3). G) Fluorescence polarization (FP) analysis of NCI-H1975 WT and OE cells after incubation with WntSI for 1, 2, and 6 hours (n = 3). The data were presented as mean ± s.d. and comparisons were performed with Student’s t-test; **, *p* < 0.01; ns. Not significant.


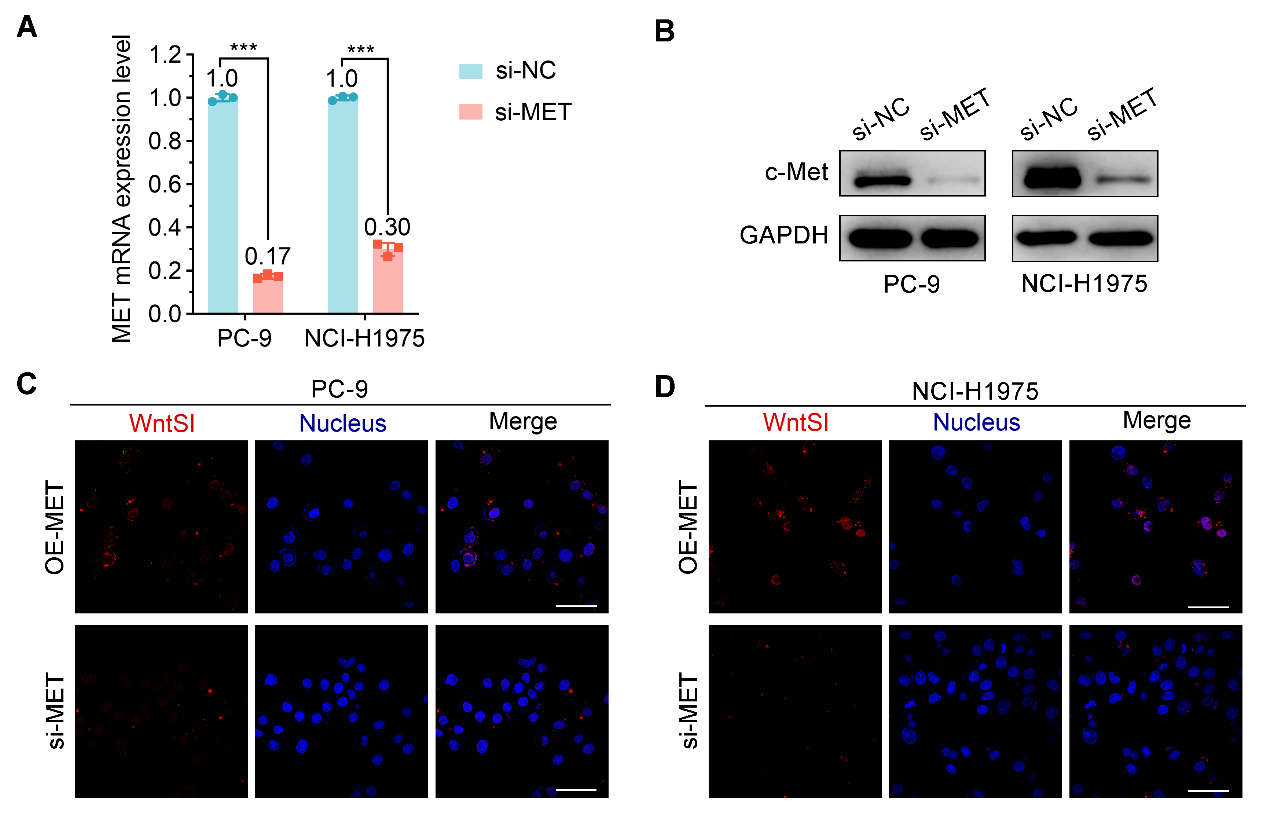


**Figure S7.** Knockdown of MET decreased the cellular uptake of WntSI in PC-9 and NCI-H1975 cells. A, B) MET mRNA (n = 3) and protein expression levels of in PC-9 and NCI-H1975 cells after transfection with MET siRNA. C, D) Laser scanning confocal microscopy (LSCM) images of cellular uptake of WntSI after overexpression and knockdown of MET in PC-9 and NCI-H1975 cells (scale bar: 50 μm). Cy5 fluorescens red was employed to label the Tyr-pep in WntSI. The data were presented as mean ± s.d. and comparisons were performed with Student’s t-test; ***, *p* < 0.001


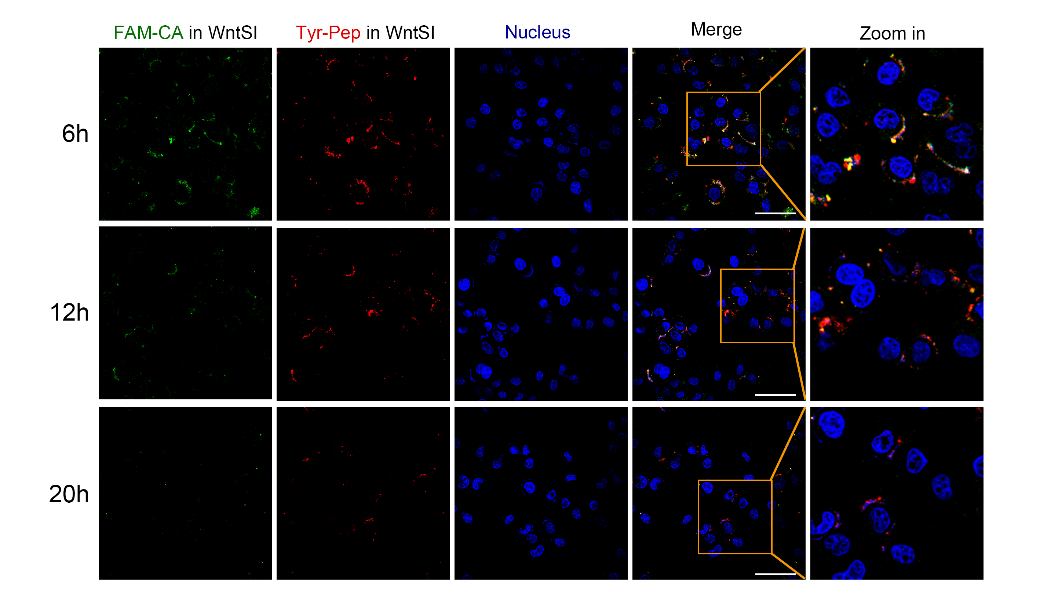


**Figure S8.** The PC-9 OE cells were incubated with double fluorescently labeled WntSI for 6 h, 12 h, and 20 h. Carboxyfluorescein (5-FAM), a fluorophore similar to CA, was utilized as a model drug in place of CA and mixed with CA at a ratio of 1:9. Cy5 fluorescens red was employed to label the Tyr-pep in WntSI. The images were captured using laser scanning confocal microscopy (LSCM) (scale bar: 50 μm).


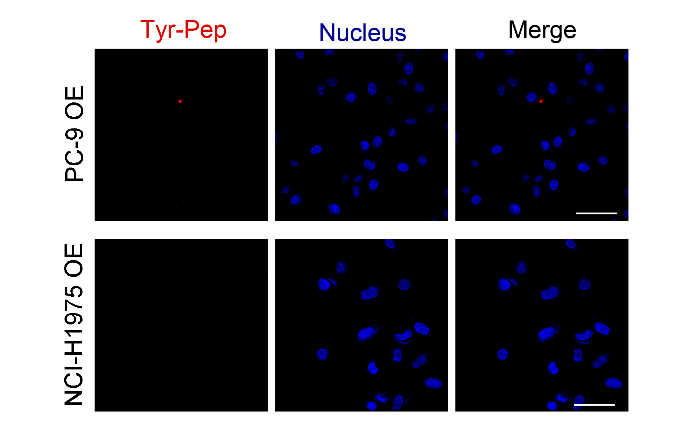


**Figure S9.** Laser scanning confocal microscopy (LSCM) images of cellular uptake of Tyr-Pep in PC-9 OE and NCI-H1975 OE cells (scale bar: 50 μm).


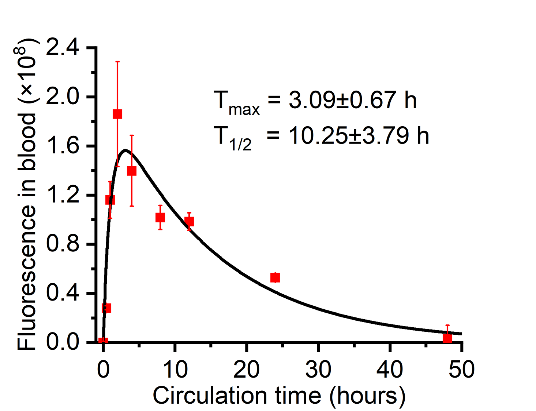


**Figure S10.** A microplate reader detected and quantified the pharmacokinetics of Cy5 SE-labeled WntSI in the blood plasma extracted from healthy C57BL/6 mice (excitation: 649 nm, emission: 670 nm) (n = 3). The data were presented as mean ± s.d.


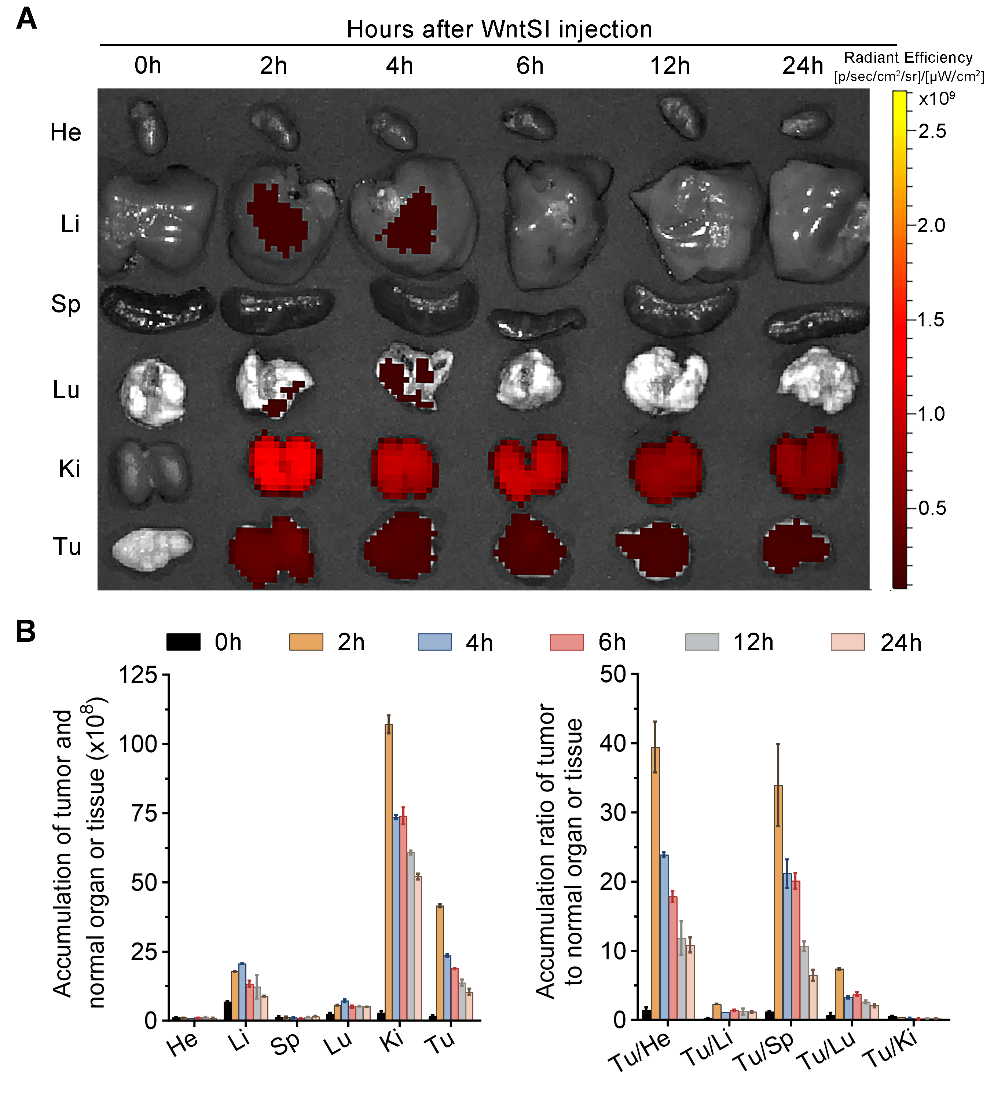


**Figure S11.** Ex vivo fluorescent images (A) and analysis (B) of major organs from Cy5 SE-labeled WntSI treated mice at 0 h, 2 h, 4 h, 6 h, 12 h, and 24 h postinjection (n = 3). He, heart; Li, liver; Sp, spleen; Lu, lung; Ki, kidney; Tu, tumor. The data were presented as mean ± s.d.


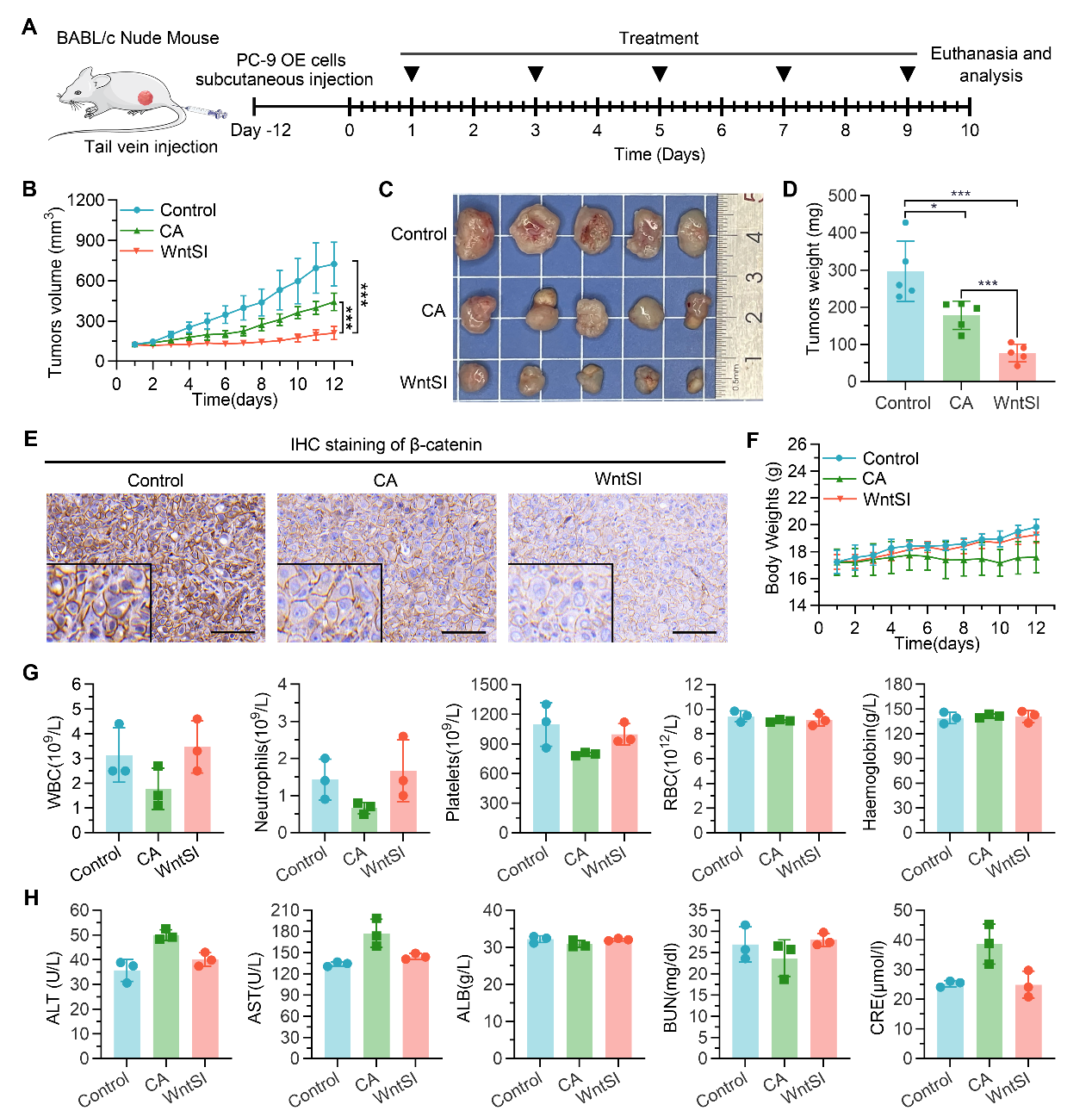


**Figure S12.** Antitumor therapeutic effect of WntSI in vivo. A) Schematic diagram of the drug intervention protocol. B) Tumor growth curve of mice in control, CA, and WntSI groups (n = 5). C) Photographs of tumor in mice after treatments. D) Tumor weight of mice in each group after treatments (n = 5). E) Immunohistochemistry (IHC) of β-catenin in representative tumor sections after treatments (scale bar: 100 μm). F) Body weight changes in mice during control, CA, and WntSI groups (n = 5). G, H) Blood routine and biochemistry examination were measured by white blood cell (WBC), neutrophils, platelets, red blood cell (RBC), hemoglobin, aspartate transaminase (ALT), alanine aminotransferase (AST), albumin (ALB), blood urea nitrogen (BUN), and creatinine (CRE) after treatments (n = 3). The data were presented as mean ± s.d. and comparisons were performed with Student’s t-test; *, *p* < 0.05; ***, *p* < 0.001


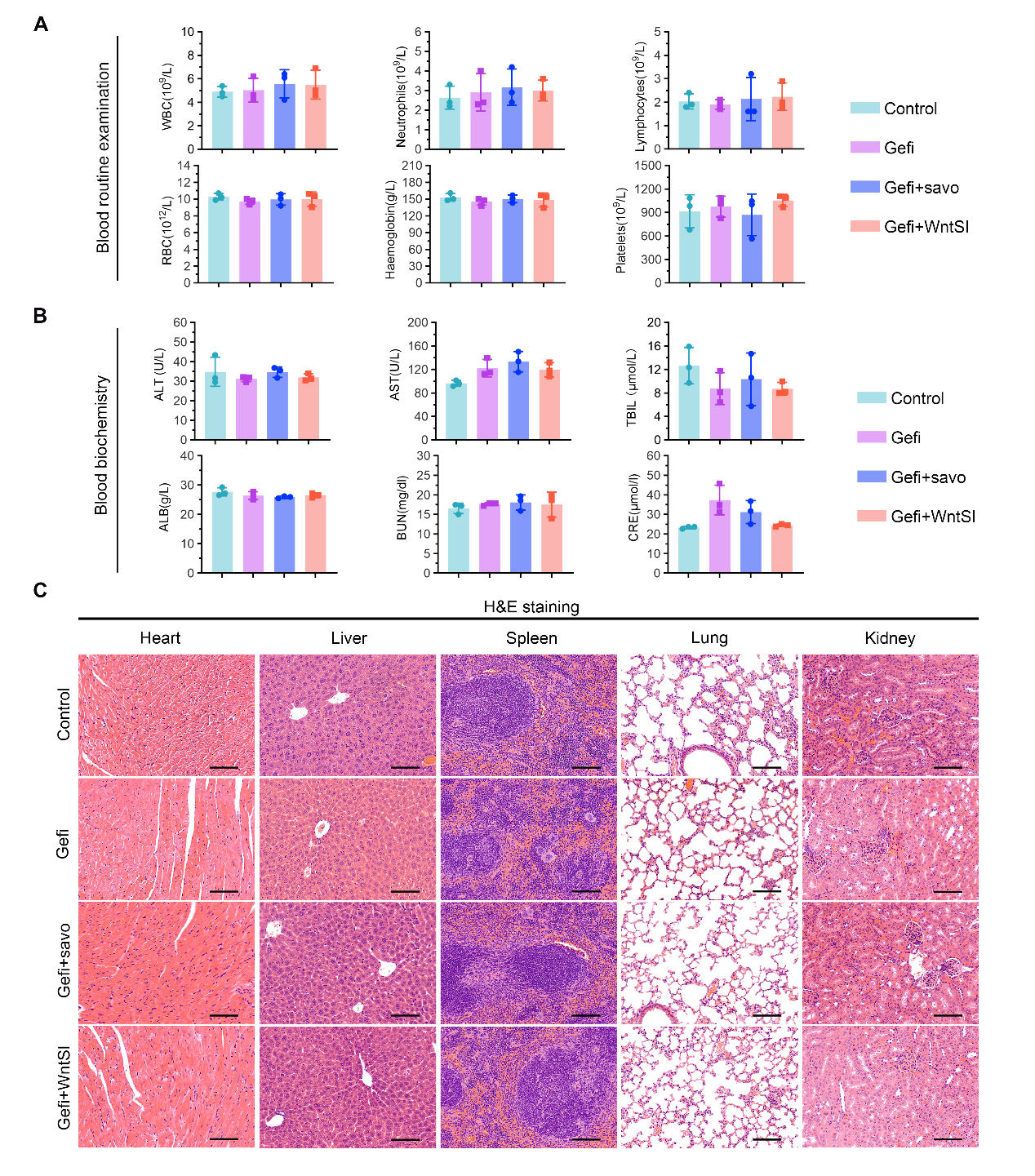


**Figure S13.** In vivo biosafety evaluation of WntSI in combination with EGFR-TKIs in PC-9 OE cell xenograft mouse model. A) White blood cell (WBC), neutrophils, lymphocyte, red blood cell (RBC), hemoglobin and platelets were examined in BALB/c nude mice blood after indicated treatments (n = 3). B) Blood biochemistry indicators ware measured by aspartate transaminase (ALT), alanine aminotransferase (AST), total bilirubin (TBIL), albumin (ALB), blood urea nitrogen (BUN), and creatinine (CRE) after indicated treatments (n = 3). C) Hematoxylin-eosin (H&E) staining of the hearts, livers, kidneys, spleens and lungs of mice after the indicated treatments (scale bar: 100 μm). The data were presented as mean ± s.d.


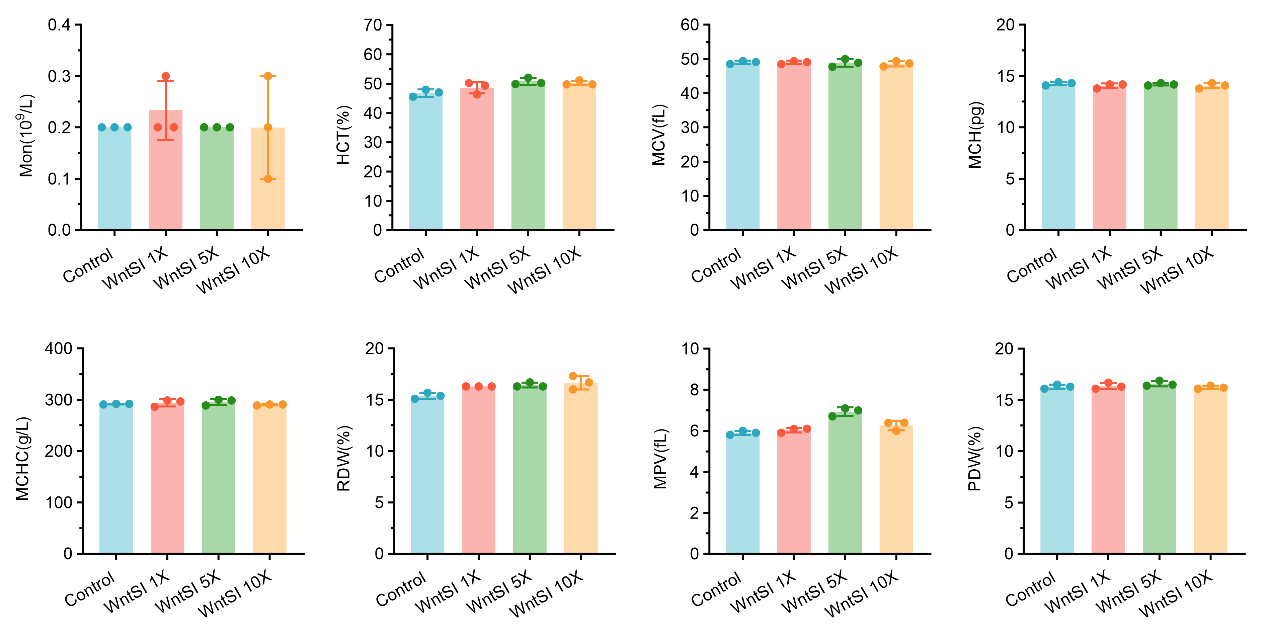


**Figure S14.** Measurement of relevant haematological indicators in C57BL/6 mice after WntSI 1× (3mg/kg), 5× (15mg/kg) and 10× (30mg/kg) dose interventions (n = 3). The data were presented as mean ± s.d.


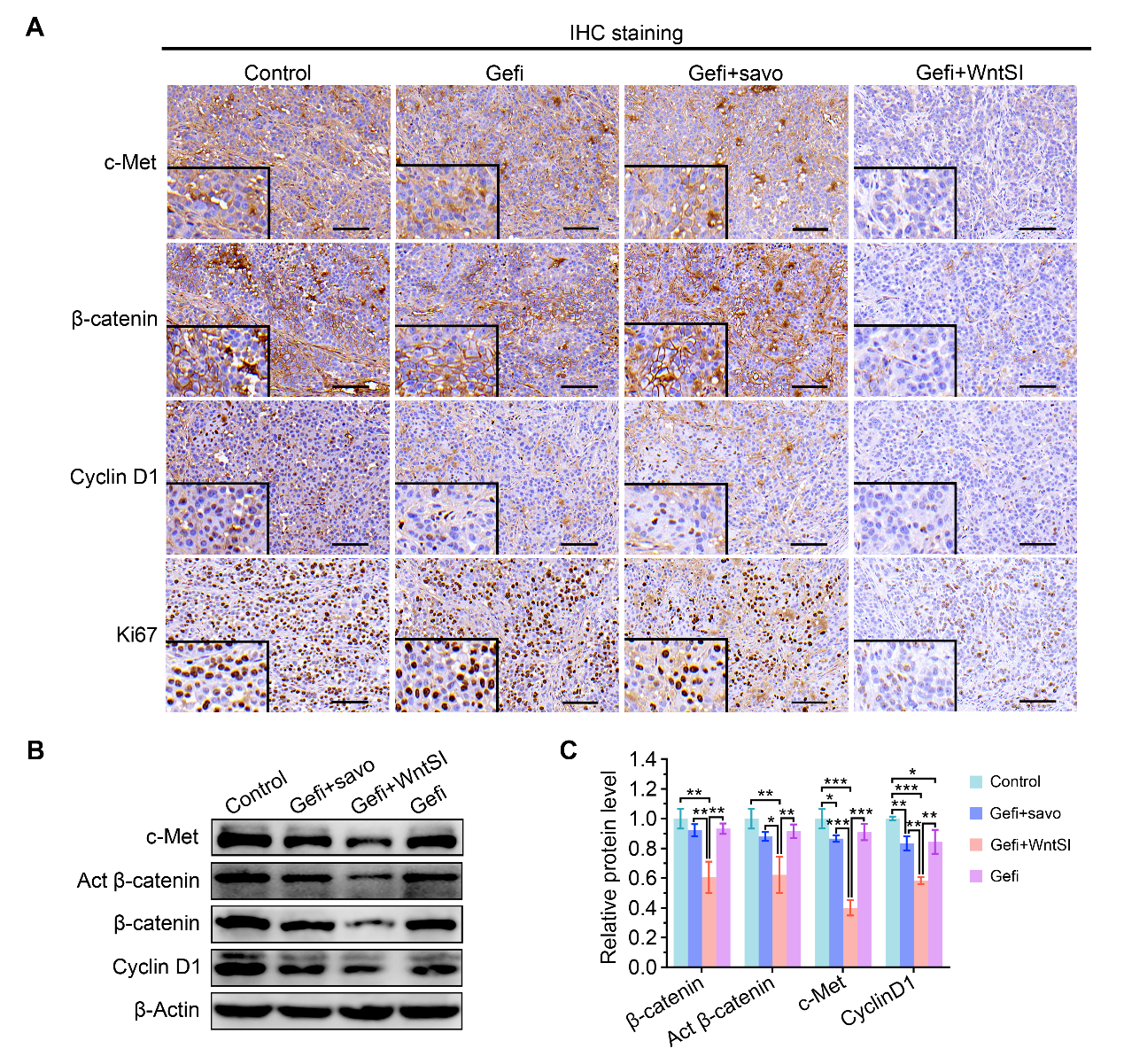


**Figure S15.** WntSI combined with EGFR-TKIs effectively inhibits the Wnt/β-catenin signaling pathway in vivo. A) Representative tumor sections after the indicated treatment staining by immunohistochemistry (IHC) of c-Met, β-catenin, Cyclin D1, and Ki67 (scale bar: 100 μm). B, C) The protein levels of Wnt/β-catenin pathway in tumor tissues of patient-derivrd xenograft (PDX) mouse model after the treatment with Gefitinib (Gefi), Gefi combined with Savolitinib (savo) or WntSI (n = 3). The data were presented as mean ± s.d. and comparisons were performed with Student’s t-test; *, *p* < 0.05; **, *p* < 0.01; ***, *p* < 0.001


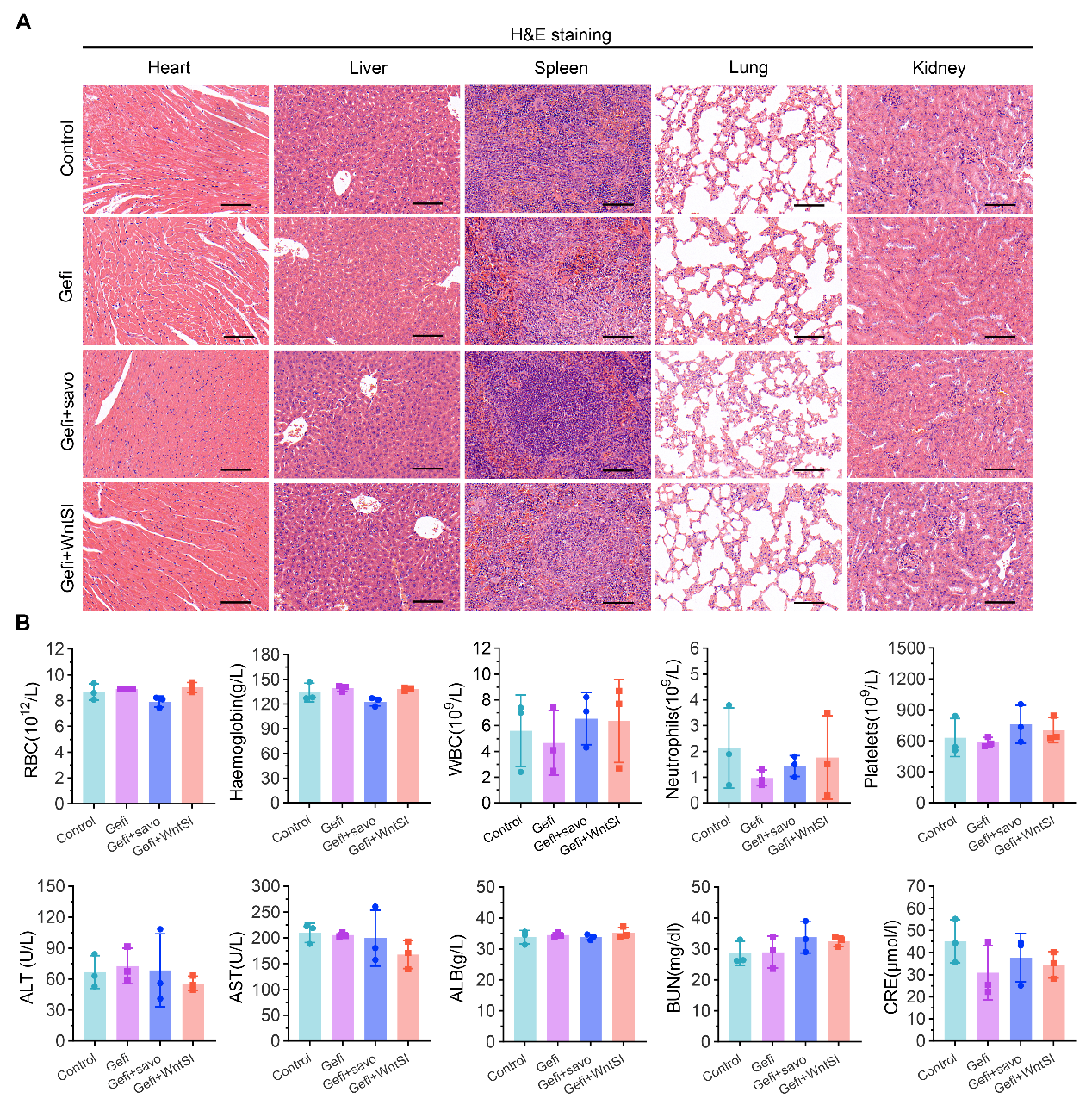


**Figure S16.** In vivo biosafety evaluation of WntSI in combination with EGFR-TKIs in NSCLC patient-derived xenograft (PDX) model. A) Hematoxylin-eosin (H&E) staining of the hearts, livers, kidneys, spleens and lungs of mice after the indicated treatments (scale bar: 100 μm). B) Blood routine and biochemistry examination were measured by red blood cell (RBC), hemoglobin, white blood cell (WBC), neutrophils, platelets, aspartate transaminase (ALT), alanine aminotransferase (AST), albumin (ALB), blood urea nitrogen (BUN), and creatinine (CRE) after indicated treatments (n = 3). The data were presented as mean ± s.d.

**2. Supplementary Table**

**Table S1.** The sequences of siRNA and primers for RT- qPCR assays.

| Gene | Sequence |
| --- | --- |
| siMET sense | GCACUAGCAAAGUCCGAGA |
| siMET antisense | UCUCGGACUUUGCUAGUGC |
| MET-F | GGGAGCCAAAGTCCTTTCAT |
| MET-R | CGAATGCAATGGATGATCTG |
| β-catenin-F | TGCTCTTCGTCATCTGACCA |
| β-catenin-R | CAATCCAACAGTAGCCTTTATCAG |
| Cyclin D1-F | CTGGAGGTCTGCGAGGAACA |
| Cyclin D1-R | AGCTGCAGGCGGCTCTTT |
| c-Myc-F | GAGCCCCTGGTGCTCCAT |
| c-Myc-R | GCCTGCCTCTTTTCCACAGA |
| Wnt2b-F | TTTCTGAAGCTGGAGTGTAAGT |
| Wnt2b-R | AAAGTAGACAAGATCAGTCCGG |
| Wnt3a-F | CAGCCACATGCACCTCAAGT |
| Wnt3a-R | GAGGCGCTGTCGTACTTGTC |
| Wnt5a-F | ATTCTTGGTGGTCGCTAGGT |
| Wnt5a-R | TGTACTGCATGTGGTCCTGA |
| Wnt7a-F | CTGTGGCTGCGACAAAGAGAA |
| Wnt7a-R | GCCGTGGCACTTACATTCC |
| Wnt7b-F | CACAGAAACTTTCGCAAGTGG |
| Wnt7b-R | GTACTGGCACTCGTTGATGC |
| Wnt10a-F | AATGCCAACACCAATTCAGG |
| Wnt10a-R | CAACTCGGTTGTTGTGAAGC |
| GAPDH-F | GGAGCGAGATCCCTCCAAAAT |
| GAPDH-R | GGCTGTTGTCATACTTCTCATGG |
